# Metagenomic and metabolomic insights into the rhizosphere of *Paeonia suffruticosa* ‘Luoyang Hong’ across a continuous cropping chronosequence

**DOI:** 10.3389/fpls.2026.1754999

**Published:** 2026-05-12

**Authors:** Kexin Sun, Fei Wang, Tongfei Niu, Hao Wang, Yining Liu, Lili Guo, Xiaohui Wang, Xiaogai Hou

**Affiliations:** 1 College of Agriculture/Tree Peony, Henan University of Science and Technology, Luoyang, China; 2 School of Plant Protection and Environment, Henan Institute of Science and Technology, Xinxiang, China; 3 Luoyang Academy of Agriculture and Forestry Sciences, Luoyang, China

**Keywords:** continuous cropping, plant-microbe interactions, rhizosphere metabolites, rhizosphere microbial community, root metabolites

## Abstract

The cultivation of *Paeonia suffruticosa* ‘Luoyang Hong’, a valuable ornamental crop, faces significant challenges due to replanting issues. However, the dynamics of its rhizosphere micro-ecosystem under continuous cropping remain poorly understood. This study systematically investigates the successional patterns of the rhizosphere micro-ecosystem over a 12- to 42-year chronosequence to identify the underlying drivers of these issues. Using an integrated multi-omics approach combining metagenomics and non-targeted metabolomics, we deciphered the rhizosphere mechanisms associated with replanting issues in *Paeonia suffruticosa* ‘Luoyang Hong’. Based on differential changes in metabolites within the soil and root systems, key substances such as succinic acid, trans-ferulic acid, vanillic acid, and Leu-Val-Arg-Lys were identified. The microbial succession demonstrated a distinct temporal progression. Initially, at the 12-year stage, the rhizosphere was enriched with beneficial bacterial genera. However, around the 20-year stage, the abundance of these beneficial genera significantly declined. Subsequently, at the 34-year stage, the community shifted to a dominance of genera associated with organic matter degradation. Finally, at the 42-year stage, a partial recovery of certain beneficial genera and their functions was observed. Despite this recovery, the overall system continued to exhibit signs of continuous degradation. Integrated multi-omics analysis further revealed significant positive correlations, such as that between N,N-dimethyldodecylamine N-oxide and several differential microbial genera, underscoring the complex interactions between metabolites and microbes. Our findings provide a systematic perspective on the micro-ecological dynamics in the rhizosphere of *Paeonia suffruticosa* ‘Luoyang Hong’, offering deeper insights into replanting issues and supporting future mitigation strategies.

## 1 Introduction

Valued for both its ecological and economic importance, the ornamental *P. suffruticosa* ‘Luoyang Hong’ is widely cultivated in northern China. However, in tree peony gardens with many years of cultivation, phenomena such as gradual decline in plant vigor, reduced flowering, frequent and difficult-to-control pests and diseases, and even large-scale plant death in severe cases, seriously restrict the sustainable development of the tree peony industry (Yang et al., 2024). Among these issues, root rot caused by soil-borne pathogens is particularly prominent (Tian et al., 2024), with a general incidence rate of about 15% in new tree peony gardens and over 30% in old tree peony gardens (Wang et al., 2012), posing a threat to both ornamental value and industrial benefits.

Allelopathic substances play an important ecological role in competing with neighboring plants, responding to pathogen infections, or avoiding herbivory, but the accumulation of these substances can cause autotoxicity, making them a “double-edged sword” for the plants themselves (Zhu et al., 2025; Fu et al., 2026), and improve the plant’s systemic disease resistance by suppressing the colonization and infection processes of pathogenic microorganisms (Kong et al., 2018), and improve the plant’s systemic disease resistance by suppressing the colonization and infection processes of pathogenic microorganisms (Ji et al., 2025). However, when these substances, such as phenolic acids, terpenoids, et al., continuously accumulate in a continuous cropping system and exceed the ecological threshold, they will in turn inhibit the vitality of the plant’s own root system, interfere with nutrient absorption, and disrupt the rhizosphere microecological balance, leading to typical autotoxicity (Xuan et al., 2024; Zhong et al., 2024). In tree peony, substances secreted by its roots, such as ferulic acid, vanillin, paeonol, and coumarin, have been confirmed to have allelopathic potentia (Qin et al., 2010; Fang et al., 2020).

Rhizosphere microorganisms, known as the second genome in plants, play an indispensable role in promoting plant growth and maintaining soil ecological balance (Wen et al., 2025). They support plant growth and development through various direct or indirect mechanisms: on the one hand, microorganisms can degrade allelochemicals such as phenolic acids and terpenoids secreted by roots, converting them into available carbon sources and energy, thereby alleviating autotoxicity (Liu et al., 2023b), on the other hand, they promote nutrient mineralization and absorption and enhance plant stress resistance by secreting phytohormones, siderophores, 1-aminocyclopropane-1-carboxylate deaminase, and other active substances (Wang et al., 2024b; Shi et al., 2024). Previous studies have shown that beneficial bacterial groups such as *Pseudomonas* and *Bacillus* exist in the rhizosphere soil of tree peony, but their abundance significantly decreases with increasing years of continuous cropping, with some beneficial bacteria even gradually disappearing (Guo et al., 2017). However, how specific allelochemicals in the rhizosphere under tree peony continuous cropping conditions affect the community dynamics and functional responses of these beneficial microorganisms is not yet clear.

Metagenomics encompasses the collective genetic material of all microorganisms in the environment, facilitating microbial species-level identification and functional predictions through high-throughput sequencing (Handelsman, 2004; Franzosa et al., 2018). Metabolomics, on the other hand, elucidates the mechanisms underlying metabolite accumulation and aids in biomarker screening (Ranner et al., 2025) The integration of metagenomics and metabolomics offers a robust methodology for analyzing the interplay between rhizosphere microecology and plant growth and development. Zhang et al. (2025) employed integrated metagenomic and metabolomic analyses to elucidate the self-repair mechanism of the soybean continuous cropping system, highlighting the feedback regulation between the rhizosphere microbiome and metabolites. Similarly, Ding et al. (2026) utilized this integrated approach to identify that the abnormal accumulation of citrulline in the rhizosphere is a critical factor driving the occurrence of Fusarium wilt.

Against this backdrop, this study takes the rhizosphere soil of *P. suffruticosa* ‘Luoyang Hong’ under different continuous cropping years (including an unplanted control) as the research object, integrating non-targeted metabolomics and metagenomic techniques to systematically investigate the long-term effects of continuous cropping on the rhizosphere ecosystem. This study focuses on the following objectives: (1) To elucidate the effects of different continuous cropping years on the key physicochemical properties of rhizosphere soil; (2) To reveal the dynamic changes of root exudates under continuous cropping conditions and identify key differential metabolites involved in allelopathy; (3) To analyze the response characteristics of the soil microbial community structure and function to the planting years; (4) To investigate the tripartite interactions of the soil environment, root metabolism, and microbial community, thereby elucidating the microecological mechanisms behind continuous cropping obstacles. This research aims to provide a theoretical basis for the ecological regulation of tree peony continuous cropping obstacles and to offer new ideas for improving tree peony cultivation quality and industrial benefits through microbial interaction strategies.

## 2 Materials and methods

### 2.1 Sample collection and determination

Samples of rhizosphere soil (SY12, SY22, SY34, SY42) and roots (RY12, RY22, RY34, RY42) from *P. suffruticosa* ‘Luoyang Hong’ were collected from 12-, 22-, 34-, and 42-year continuous cropping plots at the Resource Nursery of Henan University of Science and Technology. Unplanted soil (SY0), which had never been cultivated with tree peony, served as the control (n = 6 for each group). Soil and root samples were collected at a depth of 0–20 cm. For each sample, 10 g of root samples and 200 g of soil samples were collected. Rhizosphere soil was either air-dried for physicochemical analysis or preserved for multi-omics (metagenomics and metabolomics) study.

Soil pH was measured using the potentiometric method with a soil-to-water ratio of 1:2.5 using CO_2_-free water for extraction. Total nitrogen (TN) was determined using a combined Kjeldahl-flow injection analysis method. Total phosphorus (TP) was determined by the sodium hydroxide fusion-molybdenum antimony anti-spectrophotometry method. Total potassium (TK) was determined by the sodium hydroxide fusion-flame photometry method. NH_4_^+^-N and NO_3_^--^N were extracted with a 1 mol·L^-1^ potassium chloride solution and quantified by a flow injection analyzer. Available phosphorus (AP) was determined by sodium bicarbonate extraction-molybdenum antimony anti-spectrophotometry combined with a UV spectrophotometer. Available potassium (AK) was determined by ammonium acetate extraction-flame photometry. Soil organic matter (SOM) was determined by the potassium dichromate oxidation-sulfuric acid-mercuric sulfate catalytic method. All determination methods for these indicators followed the standards described by Shidan Bao (2000).

### 2.2 Metagenomic sequencing

Total DNA was extracted from the samples using the Fecal Genome DNA Extraction Kit (AU46111-96, BioTeke, China). The TruSeq Nano DNA LT Library Preparation Kit (Illumina, FC-121-4001) was used to construct a genomic library from the fragmented products, which was completed after end-repair, adapter ligation, index PCR amplification, and purification. The library was quantified using Qubit 1X dsDNA HS Assay Kits (Invitrogen, Q33230), and finally, paired-end sequencing was performed on the Illumina NovaSeq 6000 platform (LC Bio Technology CO., Ltd. Hangzhou, CN) according to standard procedures, with a PE150 sequencing mode and the NovaSeq 6000 XP 4-Lane Kit v1.5 (Illumina, 20043131).

Fastp software was used for quality control of the raw sequencing data, and the resulting clean reads were assembled for each sample using MEGAHIT software; based on CDS prediction results, MMseqs2 was utilized for dereplication, clustering at 95% identity and 90% coverage, and the longest sequence was selected as the representative to construct a Unigene set. Bowtie2 was used to obtain the final Unigene set for subsequent analysis and to calculate the abundance of each Unigene. DIAMOND software was used to align the Unigene protein sequences against the NR_meta database to obtain species annotation information at different taxonomic levels, and against functional databases such as KEGG and eggNOG to obtain functional annotations. Finally, the abundance information for each species and functional category was obtained by summarizing the abundance of Unigenes.

### 2.3 Metabolite extraction and analysis

Plant samples: The frozen samples were removed from the -80°C ultra-low temperature freezer and immediately placed on crushed ice for gradual thawing, and 100 mg of sample was extracted with 1 mL of pre-chilled 50% methanol, vortexed for 1 min, and incubated at room temperature for 10 min; the extract was then stored overnight at -20°C. After centrifugation at 4000 g for 20 minutes, the supernatant was transferred to a new 96-well plate. Additionally, 10 µL was taken from each extract to prepare a pooled Quality Control (QC) sample.

Soil samples: 500 mg (± 5 mg) of sample was weighed and placed in a 2.0 mL EP tube, to which 700 µL of 80% ice-cold methanol solution was added, followed by ultrasonic mixing. The mixture was then placed in a -20°C freezer for 30 min. After centrifugation at 20000 g for 15 min, 500 µL of the supernatant was transferred to another EP tube and freeze-dried. After the supernatant was freeze-dried, it was reconstituted in 100 µL of 80% ice-cold methanol solution. After centrifuging again at 20000 g for 15 min, the supernatant was transferred to an autosampler vial for UPLC-HRMS analysis. Equal volumes (10-20 µL) of extract from each sample were mixed to create a QC sample for UPLC-HRMS analysis, with all procedures performed on ice.

The liquid chromatography system used for data acquisition was the Waters ACQUITY UPLC System I Class. The analytical column used was an ACQUITY UPLC T3 (100 mm, 2.1 mm, 1.8 µm, Waters, UK).

### 2.4 Data analysis

Alpha diversity indices such as Chao1, The Observed species index, Good’s coverage, Shannon index, and Simpson index were calculated at the species level using QIIME 1; Beta diversity was visualized using Principal Coordinates Analysis (PCoA) based on Bray-Curtis distances (R language, version 3.6.0). LEfSe (Linear discriminant analysis effect size) analysis was used to identify differences between groups at various taxonomic levels, with a screening threshold of LDA>2.8 and *P* < 0.05. Spearman correlation analysis of species at the species level was performed using the ggplot2 (version 3.2.0) and ggnetwork R packages.

Metabolites were identified based on MS/MS (MS2) fragmentation spectra, with annotations assigned at Level 2 confidence (putative identification) by matching against the online KEGG and HMDB databases, and all matching scores were consistently above 70. Clustering heatmaps were generated using the pheatmap R package; PCA and analysis of significantly different metabolites were performed with the metaX R package; PLSDA analysis, including the calculation of VIP values for each variable, was conducted with the ropls R package; and correlation analysis was performed using Pearson’s correlation coefficient from the cor R package. Ultimately, significantly different metabolites were screened based on the simultaneous satisfaction of three criteria: a *P* < 0.05, a fold change>1.2, and a VIP value calculated from the PLSDA analysis. KEGG pathway enrichment analysis for differential metabolites was performed based on a hypergeometric test, with functional terms having a *P* < 0.05 considered significantly enriched.

## 3 Results

### 3.1 Changes in soil physicochemical properties with increasing continuous cropping years

Long-term cultivation of *Paeonia suffruticosa* ‘Luoyang Hong’ had a significant impact on soil physicochemical properties (*P* < 0.05) (**Table 1**). Nitrate nitrogen (NO_3_^--^N) content peaked in SY34 (17.53 ± 0.24 mg/kg), which was significantly higher than that in other treatments. Available phosphorus (AP) content showed a fluctuating trend of “increase first, then decrease, then increase again”. The AP contents in SY12 (44.61 ± 3.16 mg/kg) and SY42 (43.43 ± 1.34 mg/kg) were significantly higher than that in the control (10.52 ± 0.36 mg/kg), with SY12 being the highest, while that in SY34 (21.06 ± 0.74 mg/kg) dropped to a relatively low level. Available potassium (AK) content increased significantly after continuous cropping, peaking in SY22 (368.70 ± 6.20 mg/kg) and SY34 (363.28 ± 0 mg/kg), which were significantly higher than that in SY0 (161.61 ± 4.69 mg/kg). Although the AK content in SY42 (306.44 ± 8.12 mg/kg) decreased slightly, it was still significantly higher than the control. Soil organic matter (SOM) content showed a gradual upward trend with the extension of continuous cropping years, increasing from 15.32 ± 0.31 g/kg in SY12 to 27.22 ± 0.69 g/kg in SY42. However, the SOM content in all continuous cropping treatments was significantly lower than that in SY0 (35.01 ± 6.63 g/kg). In addition, there were no significant differences in soil pH among all treatments.

**Table 1:** Physicochemical properties of *P. suffruticosa* ‘Luoyang Hong’ rhizosphere soil under different planting years.

Soil Properties	SY0	SY12	SY22	SY34	SY42
PH	7.30 ± 0.19a	7.40 ± 0.04a	7.39 ± 0.02a	7.44 ± 0.01a	7.45 ± 0.04a
TN (g/kg)	1.89 ± 0.39ab	1.49 ± 0.09b	1.68 ± 0.24b	1.82 ± 0.22ab	2.19 ± 0.27a
TP (g/kg)	0.96 ± 0.13bc	0.70 ± 0.07c	1.00 ± 0.17bc	0.88 ± 0.06bc	1.21 ± 0.07a
TK (g/kg)	9.68 ± 0.32b	9.44 ± 0.21b	10.33 ± 0.19a	10.29 ± 0.32a	10.41 ± 0.24a
NH_4_^+^-N (mg/kg)	17.02 ± 1.26b	10.32 ± 0.46c	11.15 ± 1.33c	21.73 ± 2.13a	22.57 ± 0.94a
NO_3_^--^N (mg/kg)	5.24 ± 0.23e	12.22 ± 0.06c	11.62 ± 0.01d	17.53 ± 0.24a	12.83 ± 0.36b
AP (mg/kg)	10.52 ± 0.36d	44.61 ± 3.16a	25.67 ± 0.89b	21.06 ± 0.74c	43.43 ± 1.34a
AK (mg/kg)	161.61 ± 4.69d	177.85 ± 2.34c	368.70 ± 6.20a	363.28 ± 0a	306.44 ± 8.12b
SOM (g/kg)	35.01 ± 6.63a	15.32 ± 0.31c	21.30 ± 3.14bc	21.60 ± 0.6bc	27.22 ± 0.69bc

Data are presented as the mean ± standard deviation (mean ± SD). Different lowercase letters indicate significant differences at the level of *P* < 0.05. TN, total nitrogen; TP, total phosphorus; TK, total potassium; NH_4_^+^-N, ammonium nitrogen; NO_3_^--^N, nitrate nitrogen; AP, available phosphorus; AK, available potassium; SOM, soil organic matter.

### 3.2 Changes in metabolites in roots and rhizosphere soil with increasing years

**Figure 1:**
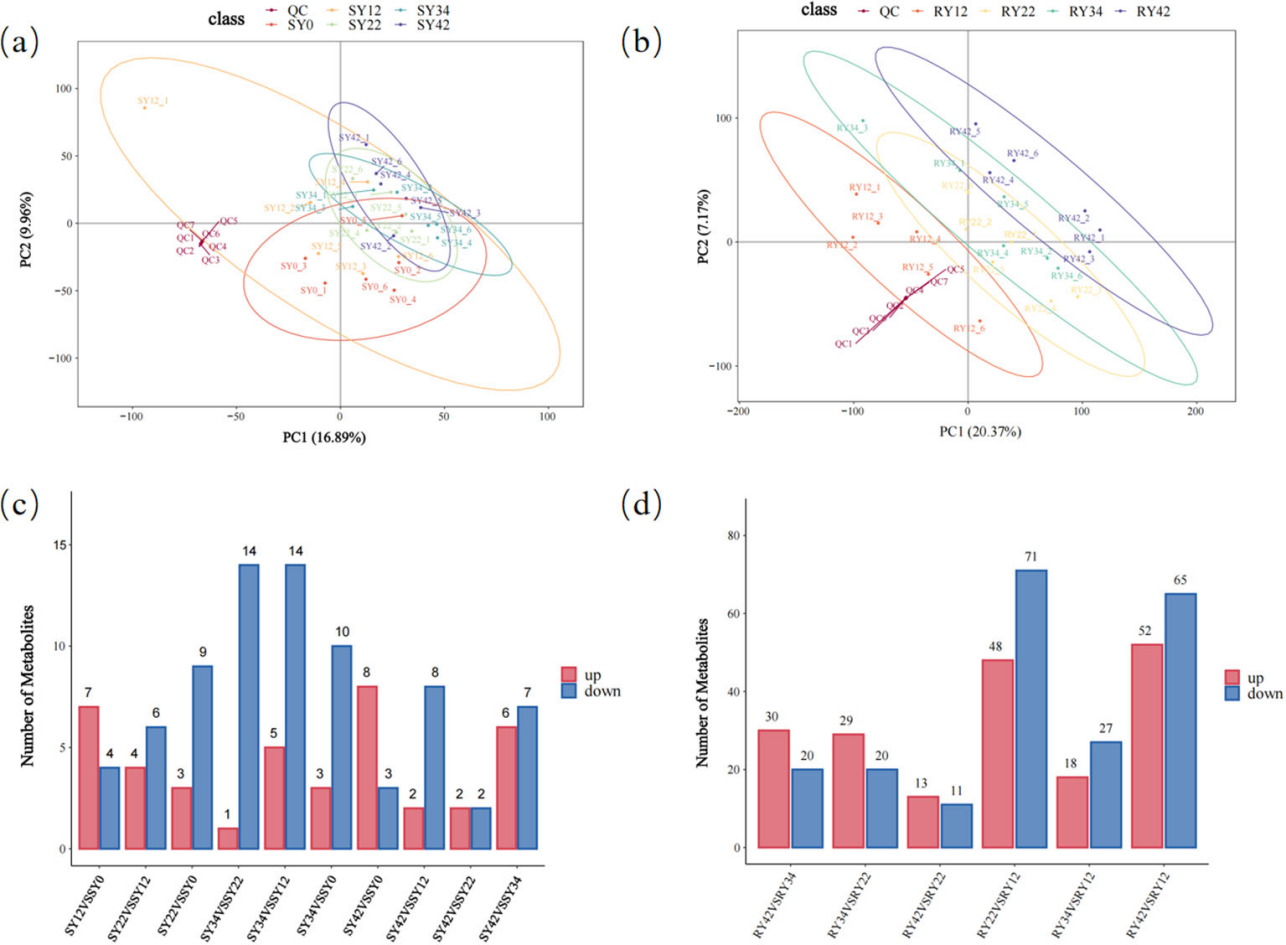
Analysis of differential metabolites in the roots and rhizosphere soil of *Paeonia suffruticosa* ‘Luoyang Hong’ under different planting years. **(a)** PLS-DA score plot of rhizosphere soil metabolites; **(b)** PLS-DA score plot of root metabolites; **(c)** Comparison of the numbers of upregulated and downregulated metabolites in each group between the two groups of rhizosphere soil metabolites; **(d)** Comparison of the numbers of upregulated and downregulated metabolites in each group between the two groups of root metabolites. Significance criteria were FC≥1.2 or FC<1/1.2, *P* < 0.05, and VIP≥1.

Based on the HMDB database, 589 metabolites were identified in the roots of *P. suffruticosa* ‘Luoyang Hong’ and 460 in the rhizosphere soil. The metabolites in the root system were classified into 11 distinct superclasses (**Supplementary Figure 1a**), including 271 species of lipids and lipid-like molecules, 73 species of phenylpropanoids and polyketides, 45 species of organic oxygen compounds, 39 species of organic acids and derivatives, 39 species of organoheterocyclic compounds, 36 species of benzenoids, 6 species of alkaloids and derivatives, 6 species of nucleosides, nucleotides, and analogues, 6 species of organic nitrogen compounds, 5 species of lignans, neolignans and related compounds, and 1 species of organosulfur compounds. Metabolites in the rhizosphere soil were classified into 12 different superclasses (**Supplementary Figure 1b**), including 211 species of lipids and lipid-like molecules, 47 species of organoheterocyclic compounds, 34 species of organic oxygen compounds, 33 species of organic acids and derivatives, 31 species of benzenoids, 20 species of phenylpropanoids and polyketides, 17 species of organic nitrogen compounds, 9 species of nucleosides, nucleotides, and analogues, 2 species of alkaloids and derivatives, 2 species of lignans, neolignans and related compounds, 1 species of homogeneous non-metal compounds, and 1 species of organophosphorus compounds. The PLS-DA plot shows varying degrees of separation for metabolites in the roots and rhizosphere soil of *P. suffruticosa* ‘Luoyang Hong’ from different planting years, with the separation being more pronounced for root metabolites, indicating a significant effect of planting year on root metabolites (**Figures 1a, b**). Among the root metabolites, the comparison group RY22VSRY12 produced the highest number of differential metabolites, while in the rhizosphere soil, the SY34VSSY12 comparison group produced the most differential metabolites. The results of the permutation test analysis for rhizosphere soil metabolites (**Supplementary Figure 2a**) indicated a fitness index (R^2^) of 0.6038, and a model predictive ability index (Q^2^) of 0.4575. Similarly, the permutation test analysis for root metabolites (**Supplementary Figure 2b**) revealed a fitness index (R^2^) of 0.6381, and a model predictive ability index (Q^2^) of -0.4512. When the horizontal axis is confined to the interval [0,1], if the R^2^ regression line is positioned above the Q^2^ regression line and the intercept of the Q^2^ regression line on the y-axis is less than 0, it suggests that the model is not overfitted. Following 200 permutation tests, the intercept of the Q^2^ regression line with the vertical axis was found to be less than 0, thereby confirming that the model is not overfitted and validating its reliability.

**Figure 2:**
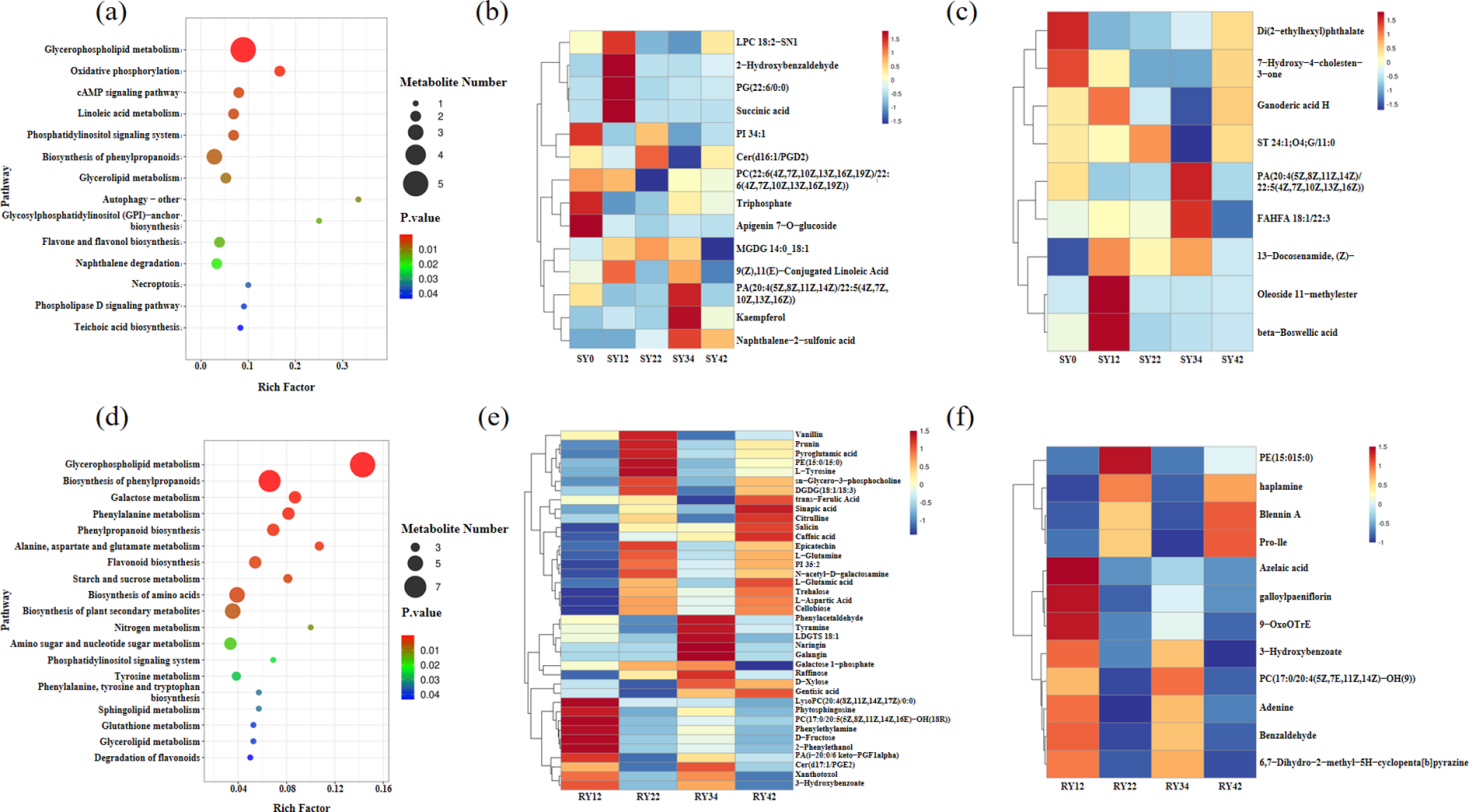
KEGG and abundance analysis of differential metabolites. **(a)** KEGG enrichment analysis of differential metabolites in rhizosphere soil; **(b)** Differential metabolites in rhizosphere soil enriched in KEGG pathways; **(c)** Differential metabolites in rhizosphere soil that appeared≥3 times in pairwise comparison groups; **(d)** KEGG enrichment analysis of differential metabolites in roots; **(e)** Differential metabolites in roots enriched in KEGG pathways; **(f)** Differential metabolites in roots that appeared≥4 times in pairwise comparison groups.

The differential metabolites identified from pairwise comparisons were subjected to KEGG pathway enrichment analysis. In the rhizosphere soil, a total of 77 differential metabolites were identified and significantly enriched in 14 metabolic pathways related to plant growth. These included glycerophospholipid metabolism, oxidative phosphorylation, biosynthesis of phenylpropanoids, glycerolipid metabolism, and flavone and flavonol biosynthesis, among others (**Figure 2a**). Among these, glycerophospholipid metabolism was enriched with the highest number of differential metabolites. These 14 pathways were enriched with a total of 14 differential metabolites, including succinic acid, 2-hydroxybenzaldehyde, kaempferol, apigenin 7-O-glucoside, etc. (**Figure 2b**). Among the ten pairwise comparison groups, nine metabolites, including beta-boswellic acid and ganoderic acid H, were identified as differential metabolites in at least three comparisons (**Figure 2c**). The relative abundance of apigenin 7-O-glucoside was highest at SY0, then declined progressively with longer cultivation of *P. suffruticosa* ‘Luoyang Hong’. The highest relative abundances of Succinic acid and 2-Hydroxybenzaldehyde were observed at SY12. The beta-boswellic acid also peaked in the SY12 group, followed by a continuous decrease through to SY42. While ganoderic acid H showed a relatively high abundance at SY12, its level dropped to a minimum at SY34. In contrast, the relative abundance of Kaempferol reached its maximum at SY34, then decreased at SY42.

In the root system, 219 differential metabolites were identified and significantly enriched in 19 plant growth-related metabolic pathways (**Figure 2d**). These pathways included glycerophospholipid metabolism, biosynthesis of phenylpropanoids, phenylalanine metabolism, flavonoid biosynthesis, starch and sucrose metabolism, and biosynthesis of amino acids. Among them, the glycerophospholipid metabolism pathway contained the highest number of enriched differential metabolites. Furthermore, a distinct set of 20 pathways was enriched for 39 differential metabolites, including trans-ferulic acid, caffeic acid, and 3-hydroxybenzoate (**Figure 2e**). Across the six pairwise comparisons, 13 compounds, such as 3-hydroxybenzoate, galloylpaeoniflorin, and azelaic acid, were detected as differential metabolites in four or more comparison groups (**Figure 2f**). Trans-ferulic acid showed an initial increasing trend, decreased at 34 years of planting, and then rebounded to its highest abundance at 42 years. Caffeic acid exhibited a consistent upward trend, reaching its peak abundance at the 42-year mark. In contrast, 3-hydroxybenzoate displayed a fluctuating pattern of increase, decrease, followed by another increase and subsequent decrease. It showed relatively high abundance at 12 and 34 years, and lower abundance at 22 and 42 years (**Figure 2e**).

**Figure 3:**
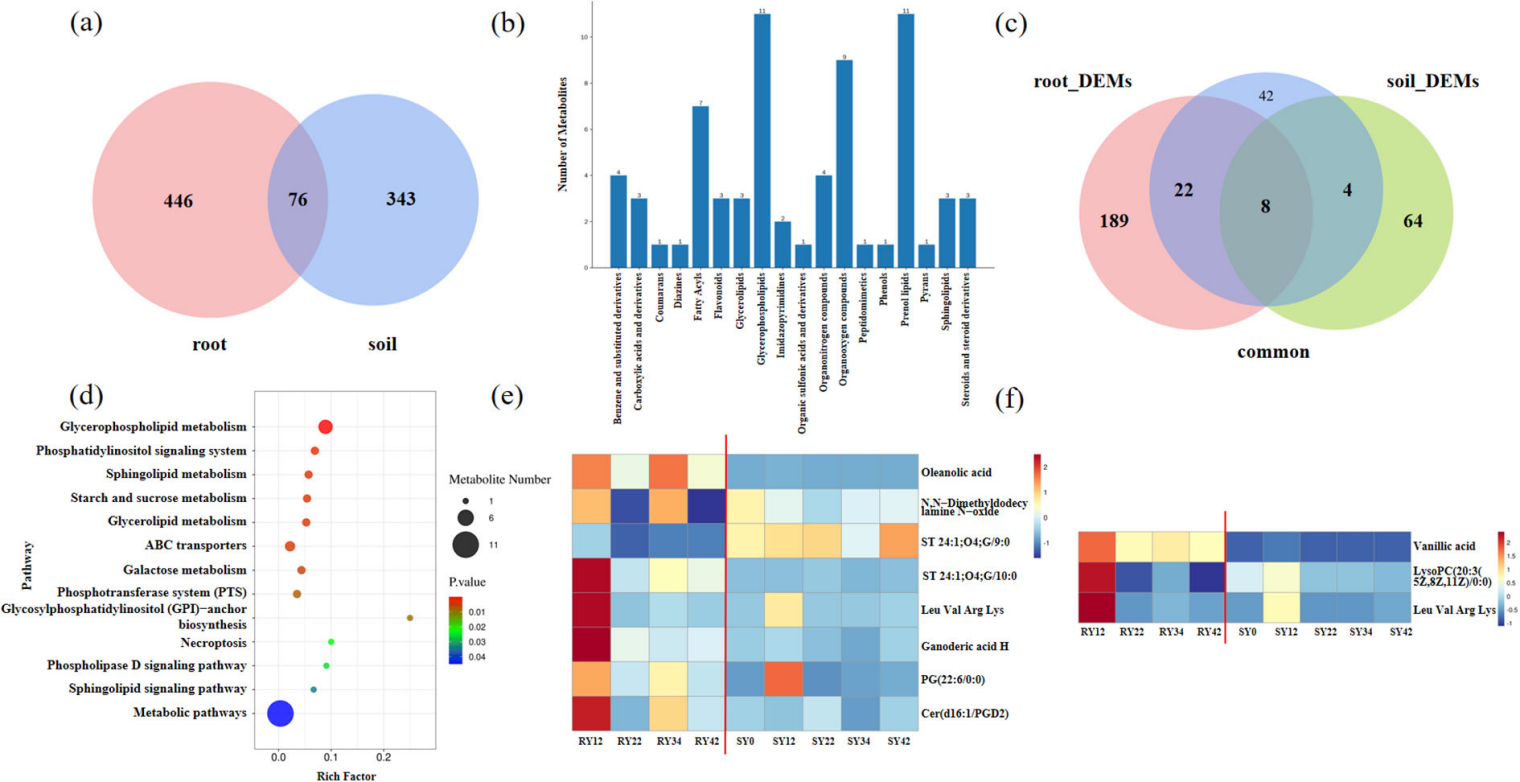
Analysis of shared metabolites between roots and rhizosphere soil. **(a)** Venn diagram of metabolites in roots and rhizosphere soil; **(b)** Classification of shared metabolites in roots and rhizosphere soil; **(c)** KEGG enrichment of shared metabolites in roots and rhizosphere soil; **(d)** Venn diagram of shared metabolites and differential metabolites in roots and rhizosphere soil; **(e)** Abundance of shared and differentially expressed metabolites in soil and roots; **(f)** Abundance of significantly correlated differential metabolites in soil and roots. ‘common’ refers to metabolites shared by roots and rhizosphere soil; ‘root_DEMs’ refers to differential metabolites generated from pairwise comparisons in roots; ‘soil_DEMs’ refers to differential metabolites generated from pairwise comparisons in rhizosphere soil. These three groups were used to create the Venn diagram.

A total of 76 common metabolites were identified across both the root system and the rhizosphere soil (**Figure 3a**; **Supplementary Figure 3**). These metabolites were categorized into 18 classes, including prenol lipids and glycerophospholipids. Metabolites belonging to the prenol lipids and glycerophospholipids classes were the most abundant (**Figure 3b**). A subset of 34 metabolites, which were common to both the root system and rhizosphere soil and showed significant differences in at least one of these compartments, was identified (**Figure 3c**; **Supplementary Figure 4**). These metabolites were enriched in 13 related pathways: Glycerophospholipid metabolism, phosphatidylinositol signaling system, sphingolipid metabolism, Starch and sucrose metabolism, glycerolipid metabolism, ABC transporters, galactose metabolism, phosphotransferase system (PTS), glycosylphosphatidylinositol (GPI)-anchor biosynthesis, necroptosis, phospholipase D signaling pathway, sphingolipid signaling pathway, and metabolic pathways (**Figure 3d**). There were 8 common differential metabolites shared between the root system and the soil, including oleanolic acid, N,N-dimethyldodecylamine N-oxide, Leu-Val-Arg-Lys, ganoderic acid H, among others (**Figure 3e**). Based on the correlation analysis of common metabolites between soil and root systems, it was found that the abundance changes of vanillic acid, lysoPC(20:3(5Z,8Z,11Z)/0:0), and Leu-Val-Arg-Lys in the soil showed a significant positive correlation with their abundance changes in the root systems (**Supplementary Figure 3**). In the soil, these three metabolites peaked at 12 years of planting, followed by a continuous decline to their lowest abundance at 34 years, with a slight recovery observed at 42 years. In the root system, all three metabolites also exhibited their highest abundance at 12 years, after which their levels decreased to varying degrees (**Figure 3f**).

### 3.3 Composition and diversity of rhizosphere microbial communities of *P. suffruticosa* ‘Luoyang Hong’ under different planting years

**Figure 4:**
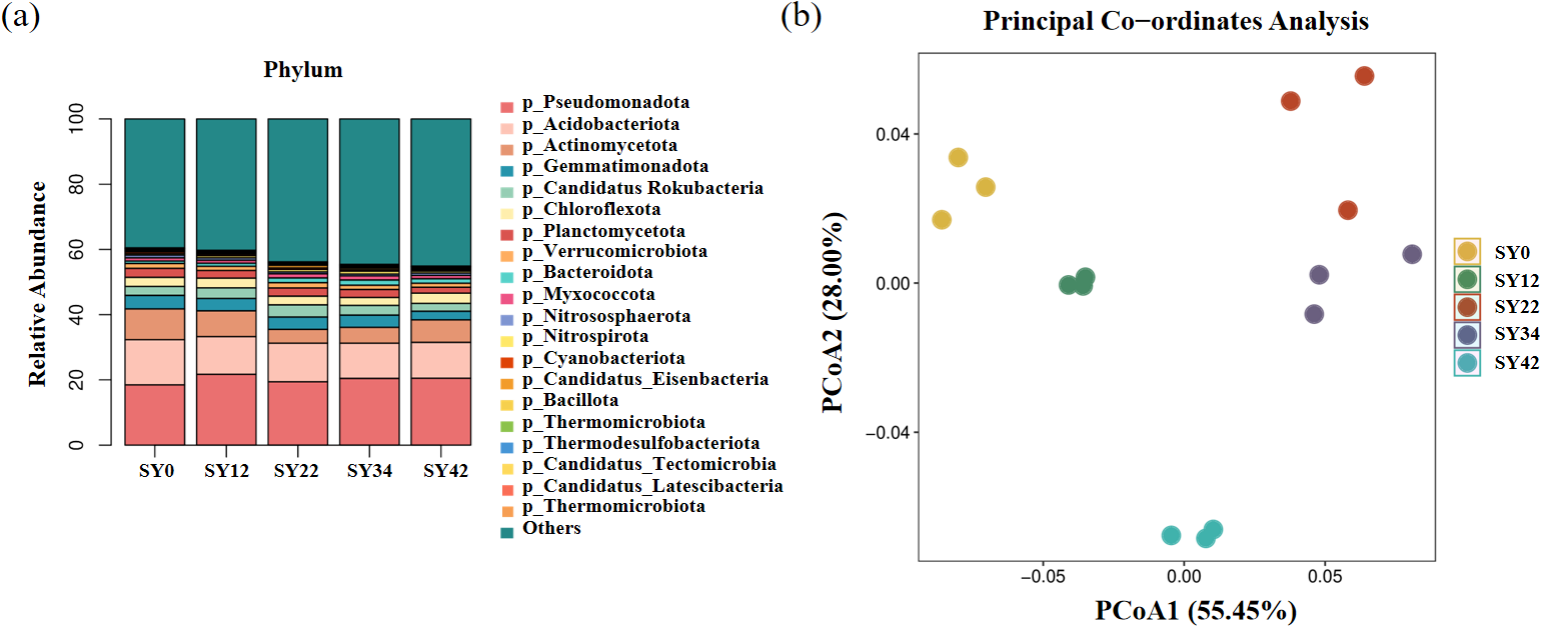
Diversity and composition of rhizosphere microbial communities. **(a)** PCoA of rhizosphere microbial community OTUs; **(b)** Phylum-level composition of rhizosphere soil microbial communities.

The results showed that continuous cropping had no effect on the alpha diversity of the microbial community (**Table 2**). Principal coordinate analysis based on Bray-Curtis distance revealed a significant separation of microbial communities across the SY0, SY12, SY22, SY34, and SY42 groups, indicating that continuous cropping restructured the microbial community (**Figure 4a**).

**Table 2:** Changes in α-diversity of microbial communities with planting time.

Treatment	Shanno	goods_coverage	Simpson
SY0	6.54 ± 0.04a	1.00 ± 0a	0.89 ± 0a
SY12	6.64 ± 0.04a	1.00 ± 0a	0.88 ± 0a
SY22	6.46 ± 0.15a	1.00 ± 0a	0.87 ± 0a
SY34	6.49 ± 0.05a	1.00 ± 0a	0.86 ± 0a
SY42	6.29 ± 0.02a	1.00 ± 0a	0.84 ± 0a

Data are presented as the mean ± standard deviation (mean ± SD). Different lowercase letters indicate significant differences at the level of *P* < 0.05.

A total of 5 kingdoms, 208 phyla, 388 classes, 669 orders, 1,360 families, 4,873 genera, and 33,885 species were identified in the rhizosphere soil samples (**Figure 4b**). At the phylum level, *p_Pseudomonadota* had the highest abundance, accounting for 18.50%, 21.71%, 19.42%, 20.48%, and 20.51% of the SY0, SY12, SY22, SY34, and SY42 groups, respectively. *p_Acidobacteriota* was the phylum with the second-highest abundance, representing 13.82%, 11.57%, 11.84%, 10.78%, and 11.01% of the five groups, respectively.

**Figure 5:**
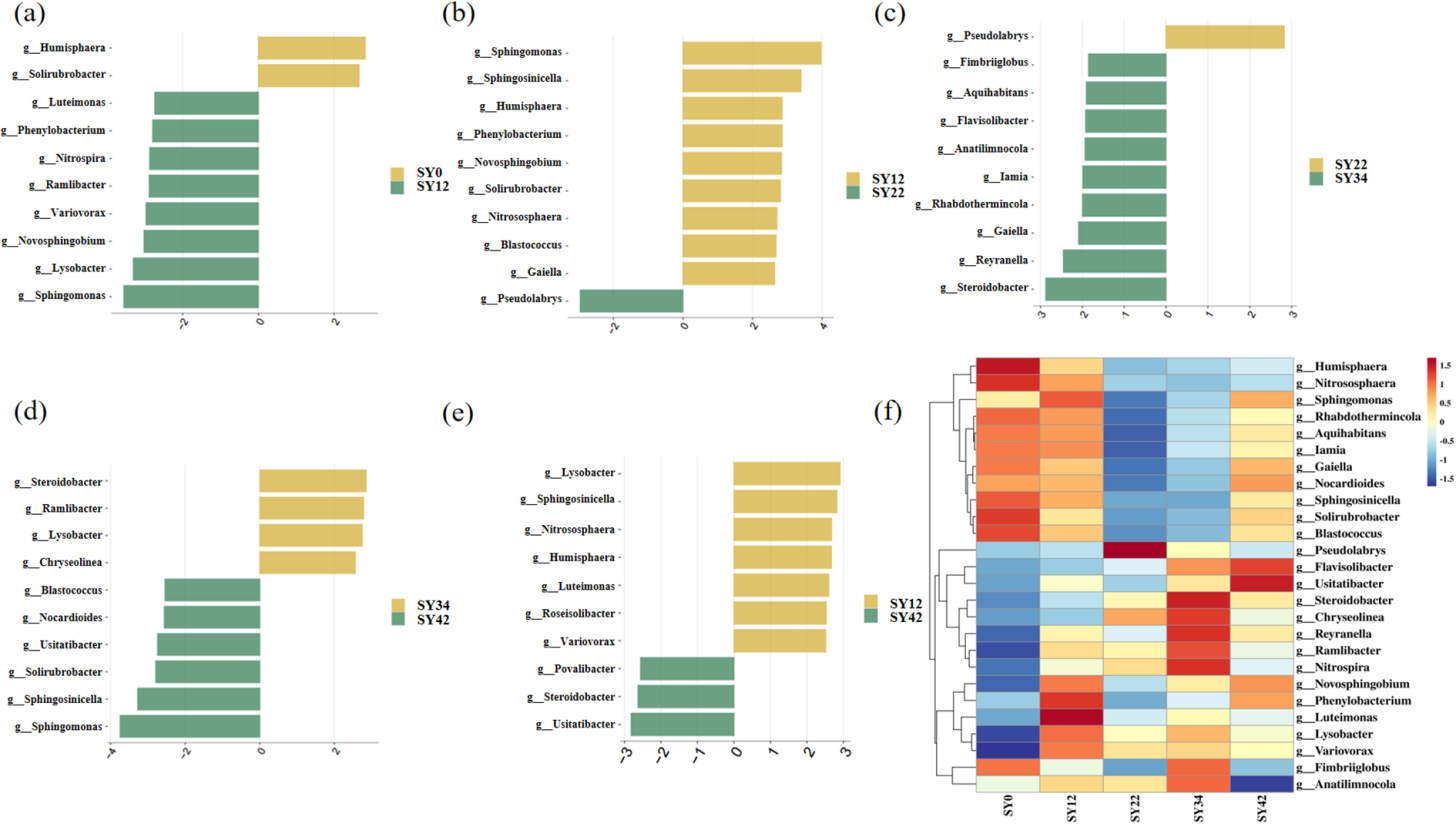
LEfSe analysis identified the differential microbial genera. **(a)** Differential microbial genera from the comparison of SY12 versus SY0; **(b)** Differential microbial genera from the comparison of SY22 versus SY12; **(c)** Differential microbial genera from the comparison of SY34 versus SY22; **(d)** Differential microbial genera from the comparison of SY42 versus SY34; **(e)** Differential microbial genera from the comparison of SY42 versus SY12; **(f)** Abundance changes of the differential microbial genera identified through pairwise comparison between adjacent groups. LDA score>2.8 and *P* < 0.05. The top 10 genera with significant differences are displayed.

LefSe analysis identified key genera associated with the continuous cropping duration of *P. suffruticosa* ‘Luoyang Hong’ (**Figure 5**). Compared to the control group, the SY12 group exhibited a significant enrichment of Sphingomonas, Lysobacter, Novosphingobium, Variovorax, Ramlibacter, Nitrospira, Phenylobacterium, and Luteimonas, whereas the relative abundances of Humisphaera and Solirubrobacter were significantly reduced (**Figure 5a**).

In the transition from SY12 to SY22, Pseudolabrys became enriched, while the abundances of Sphingomonas, Sphingosinicella, Humisphaera, Phenylobacterium, Novosphingobium, Solirubrobacter, Nitrososphaera, Blastococcus, and Gaiella decreased significantly (**Figure 5b**). Further shifts were observed between SY22 and SY34, with significant enrichment of Steroidobacter, Reyranella, Gaiella, Rhabdothermincola, Iamia, Anatilimnocola, Flavisolibacter, Aquihabitans, and Fimbriiglobus, alongside a marked decline in Pseudolabrys (**Figure 5c**).

When comparing SY34 to SY42, genera such as Sphingomonas, Sphingosinicella, Solirubrobacter, Usitatibacter, Nocardioides, and Blastococcus were enriched. Conversely, the abundances of Steroidobacter, Ramlibacter, Lysobacter, and Chryseolinea decreased (**Figure 5d**).

Finally, relative to the SY12 group, SY42 was characterized by the enrichment of Usitatibacter, Steroidobacter, and Povalibacter, and a significant depletion of Lysobacter, Sphingosinicella, Nitrososphaera, Humisphaera, Luteimonas, Roseisolibacter, and Variovorax (**Figure 5e**).

### 3.4 Microbial function analysis

**Figure 6:**
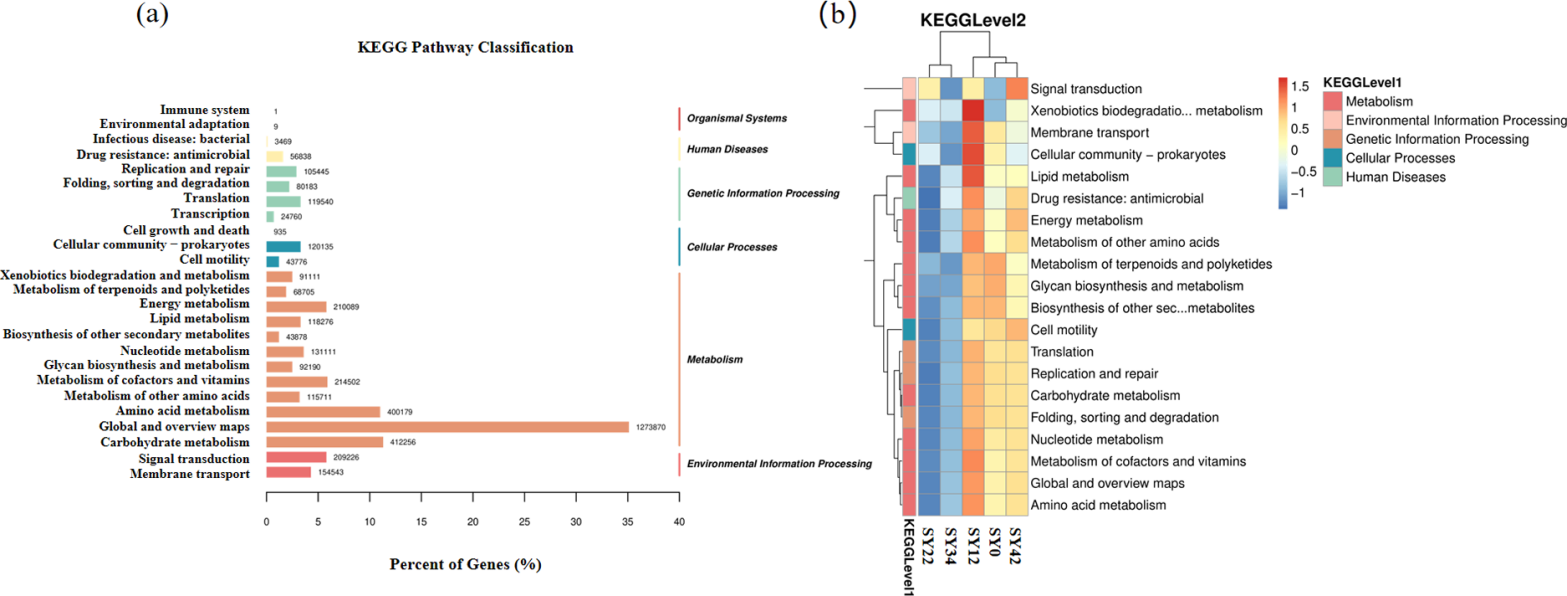
Microbial functions in the rhizosphere of *Paeonia suffruticosa* ‘Luoyang Hong’ under different cultivation years. **(a)** KEGG Pathway Classification; **(b)** Variations in microbial relative abundance across the Top 20 KEGG Level 2.

Microbial potential functional prediction for the rhizosphere soil of *P. suffruticosa* ‘Luoyang Hong’ was conducted based on metagenomic data using the KEGG database. At KEGG Level 1, Metabolism was the pathway with the highest number of annotated genes. At KEGG Level 2, the dominant predicted pathways across all samples included global and overview maps, carbohydrate metabolism, amino acid metabolism, energy metabolism, and nucleotide metabolism (**Figure 6a**). Among the top 20 KEGG Level 2 pathways ranked by relative abundance, the potential functional profiles of the microbial community were highly similar among the 0-, 12-, and 42-year continuous cropping groups, and also shared high similarity between the 22- and 34-year groups. Specific potential pathways, including Carbohydrate metabolism, Amino acid metabolism, Metabolism of cofactors and vitamins, Biosynthesis of other secondary metabolites, and Metabolism of terpenoids and polyketides, all exhibited a consistent trend: they were enriched in the 0-, 12-, and 42-year groups, while their relative abundances decreased in the 22- and 34-year groups (**Figure 6b**).

**Figure 7:**
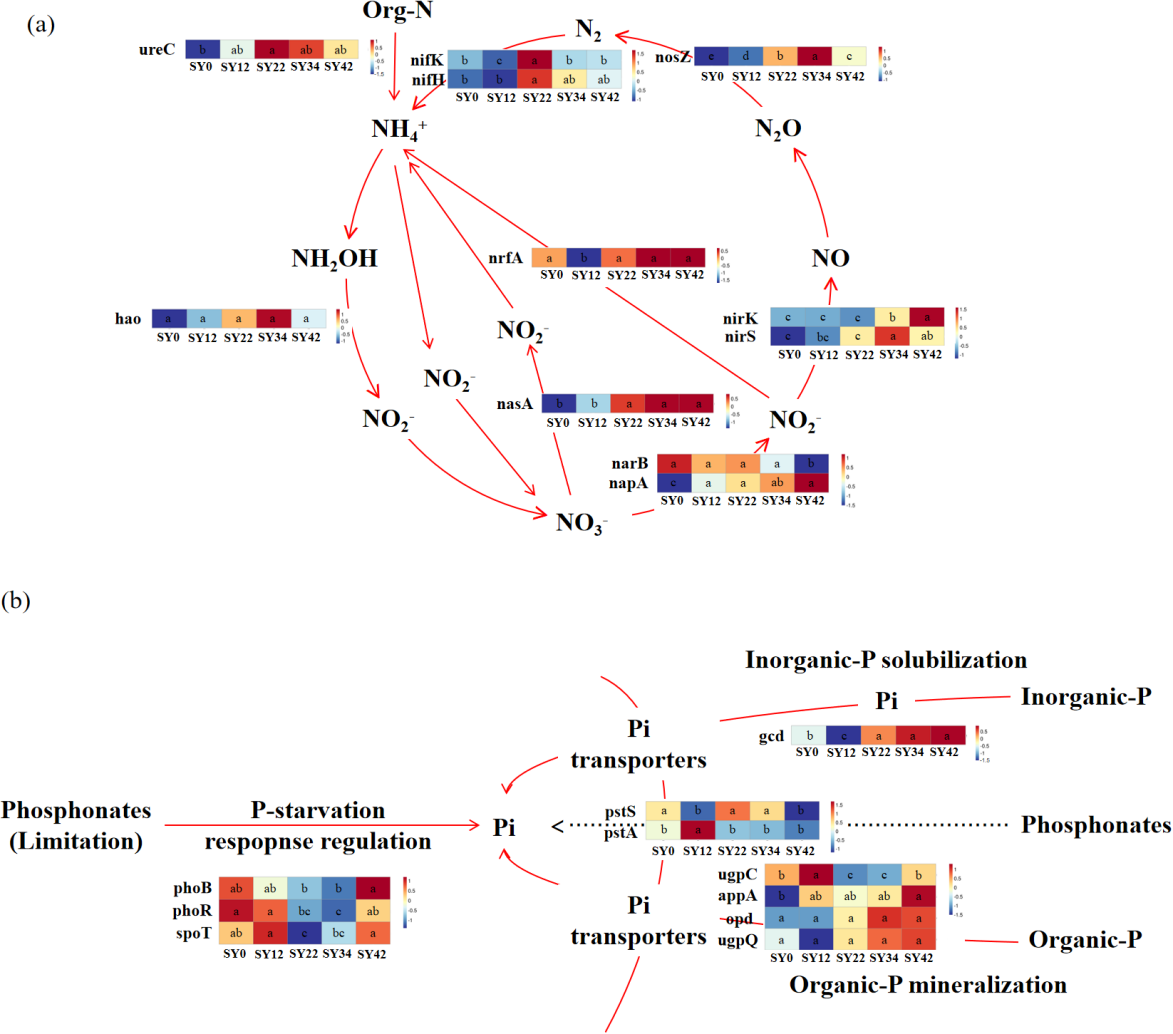
Alterations in the abundance of genes associated with nitrogen and phosphorus metabolism. **(a)** Abundance changes of phosphorus metabolism-related genes; **(b)** Abundance changes of nitrogen metabolism-related genes. Different lowercase letters indicate significant differences at the level of *P* < 0.05.

The NCycDB database was used to analyze the N-cycling gene (sub)families, and a total of 62 related genes were identified, among which 12 are directly involved in soil nitrogen cycling (**Figure 7a**). Compared with the control group (0-year), the abundance of the organic nitrogen mineralization gene (*ureC*) increased significantly in the 12-, 22-, 34-, and 42-year groups; the abundance of *nasA* increased with the extension of planting time; the abundances of nitrogen fixation genes (*nifK*, *nifH*) reached the highest at 34 years of planting. When the PCycDB database was used to analyze the P-cycling gene (sub)families, a total of 138 related genes were identified, among which 12 are directly involved in soil phosphorus cycling (**Figure 7b**). Compared to the control group (0-year), the organic phosphorus mineralization gene *appA* was significantly increased in the 12-, 22-, 34-, and 42-year continuous cropping groups. Compared to the control group (0-year), the inorganic phosphorus solubilizing gene (*gcd*) was significantly increased in the 22-, 34-, and 42-year groups. Compared with the control group (0-year), the abundances of P-starvation response regulation genes (*phoB*, *phoR*, *spoT*) decreased significantly in the 22-year and 34-year groups; in the process of Pi transporters, compared with the control group (0-year), the abundance of the *pstA* gene decreased significantly in the 22-year, 34-year, and 42-year groups, while the abundance of the *pstS* gene decreased significantly in the 12-year and 42-year groups.

### 3.5 Correlations among soil nutrients, soil metabolites, soil microbial community, and functions

**Figure 8:**
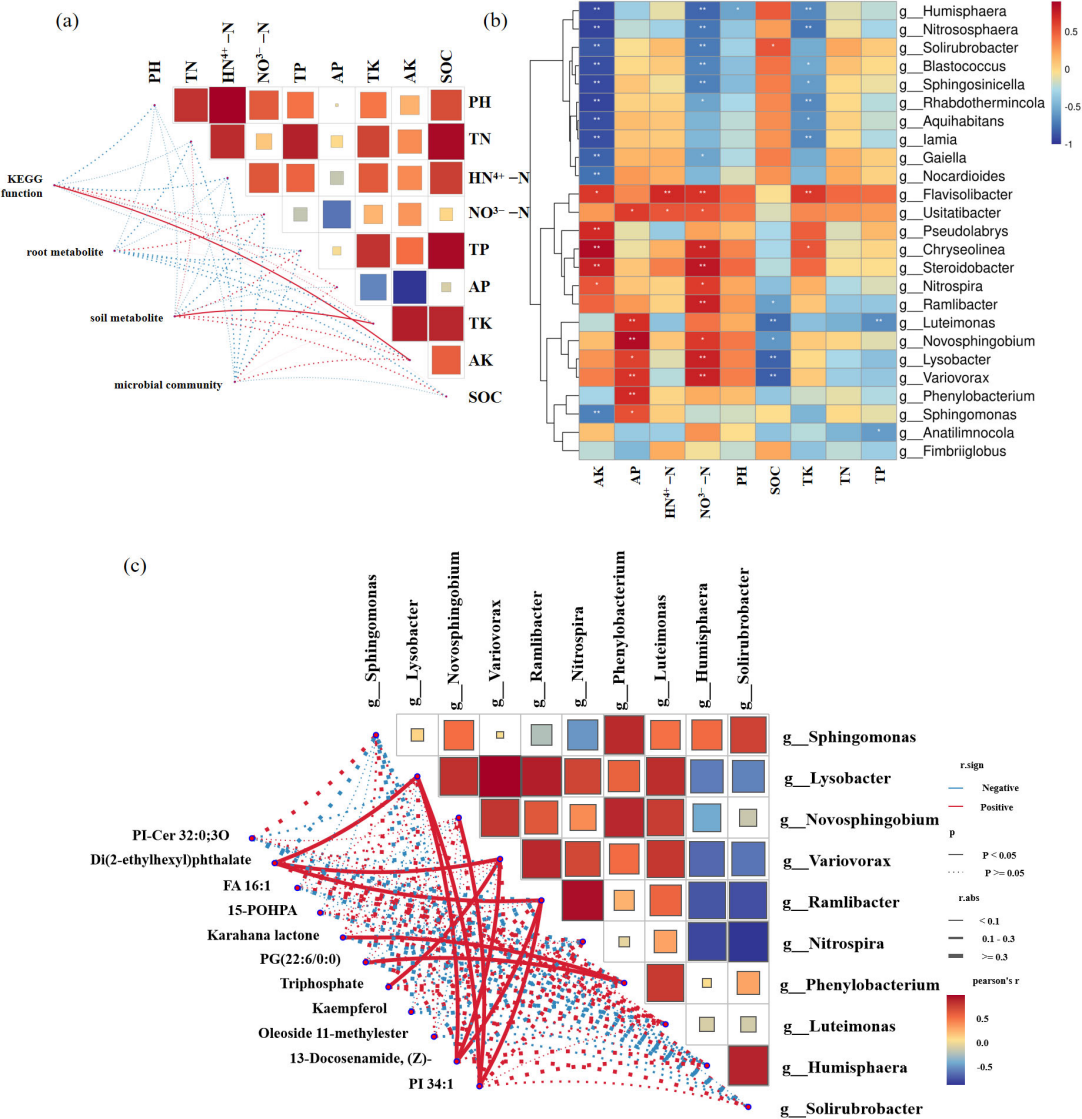
Correlation analysis. **(a)** Mantel test of soil nutrients, microbial community, KEGG functions, soil metabolites, and root metabolite composition; **(b)** Correlation between soil physicochemical properties and differential microbial genera; **(c)** Correlation between shared differential metabolites in roots and soil and differential microbial genera. Pearson correlation test was used (*P ≤* 0.05). In the heatmap on the right, the colors of the squares represent the strength of correlation between metabolite groups: the redder the color, the stronger the positive correlation; the bluer the color, the stronger the negative correlation. The network diagram in the bottom left corner shows the correlation analysis results between differential species and differential metabolites, where the thickness of the lines represents the r-value (the thicker the line, the stronger the correlation), red lines indicate positive correlation, blue lines indicate negative correlation, solid lines indicate *P<*0.05, and dashed lines indicate *P*≥0.05. *indicates *P<*0.05, and **indicates *P<*0.01.

Mantel test showed that TK was significantly positively correlated with rhizosphere soil metabolites; available potassium (AK) significantly affected the KEGG functional composition and was positively correlated; SOM was significantly positively correlated with the soil metabolite composition (**Figure 8a**).

These differential microorganisms were mostly significantly correlated with AK, NO_3_^--^N, TK, and AP. *Flavisolibacter* was significantly positively correlated with soil AK, NH_4_^+^-N, NO_3_^--^N, and TK; *Humisphaera* was significantly negatively correlated with soil AK, NO_3_^--^N, pH, and TK (**Figure 8b**).

In the comparison group of SY0 and SY12, *Lysobacter* showed a significant positive correlation with Di(2-ethylhexyl)phthalate, 13-Docosenamide, (Z)-, and PI 34:1; *Novosphingobium* showed a significant positive correlation with PI 34:1; *Variovorax* showed a significant positive correlation with Di(2-ethylhexyl)phthalate, Triphosphate, and 13-Docosenamide, (Z)-; *Ramlibacter* showed a significant positive correlation with Di(2-ethylhexyl)phthalate and 13-Docosenamide, (Z)-; *Nitrospira* showed a significant positive correlation with 13-Docosenamide, (Z)-; *Phenylobacterium* showed a significant positive correlation with Karahana lactone, PG(22:6/0:0), and Oleoside 11-methylester (**Supplementary Figure 7a**). In the comparison group of SY12 and SY22, *Phenylobacterium* showed a significant positive correlation with Oleanolic acid, PG (22:6/0:0), Oleoside 11-methylester, and Octadeca-9,12-dieneperoxoic acid (**Supplementary Figure 7b**). In the comparison group of SY22 and SY34, *Fimbriiglobus* showed a significant positive correlation with Hypogeic acid and ST 24:1;O4;G/10:0 (**Supplementary Figure 7c**). In the comparison group of SY34 and SY42, 13-Docosenamide, (Z)- showed a significant positive correlation with *Ramlibacter* and *Lysobacter*. And Ganoderic acid H is significantly positively correlated with *Chryseolinea* (**Supplementary Figure 7d**).

Among the 8 differential metabolites in roots and rhizosphere soil, N,N-Dimethyldodecylamine N-oxide showed a significant positive correlation with most differential microbial genera, including *Humisphaera*, *Solirubrobacter*, *Sphingosinicella*, *Blastococcus*, etc.; *Phenylobacterium* showed a significant positive correlation with Oleanolic acid, Leu-Val-Arg-Lys, and PG(22:6/0:0); Ganoderic acid H showed a significant positive correlation with *Chryseolinea*; *Fimbriiglobus* showed a significant positive correlation with ST 24:1;O4;G/9:0 and ST 24:1;O4;G/10:0 (**Figure 8c**).

A correlation analysis between functional genes and soil differential metabolites was conducted (**Supplementary Figure 3**). Among the genes related to nitrogen metabolism, *nrfA* showed a significant positive correlation with the abundance of Isoflavonoids, but was negatively correlated with Coumarins and derivatives, Lactones, Organooxygen compounds, Epoxides, Prenol lipids, Carboxylic acids and derivatives, and Fatty Acyls. The *nrf* gene was positively correlated with the abundances of Isoflavonoids, Indoles and derivatives, and Pyrenes. Similarly, *nifH* exhibited significant positive correlations with Isoflavonoids and Pyrenes. In contrast, the *amoC_B* gene demonstrated a positive correlation with Organic sulfonic acids and derivatives and Steroids and steroid derivatives, while showing a negative correlation with Glycerolipids. Among phosphorus metabolism-related genes, the abundance of *pstA* was significantly negatively correlated with Isoflavonoids, and significantly positively correlated with Lactones, Organooxygen compounds, Epoxides, Prenol lipids, Carboxylic acids and derivatives, and Fatty Acyls (**Supplementary Figure 4**).

## 4 Discussion

### 4.1 Continuous cropping changed the composition of soil metabolites

When plants are subjected to stress and respond to environmental pressures, their root exudate profiles typically undergo changes. The autotoxicity caused by these root exudates is considered one of the primary causes of continuous cropping obstacles in crops. In the cases of *Panax notoginseng* (Xiang et al., 2022), *Rehmannia glutinosa* (Zhang et al., 2020), *Medicago sativa* (Ma Y. Y. et al., 2024), and *Nicotiana tabacum* (Li et al., 2024a), the increase in autotoxic allelochemicals in root exudates due to continuous cropping has been identified as a key factor leading to replant failure. Succinic acid can recruit beneficial bacteria such as *Sphingomonas* to control diseases. Screening of 23 root exudates from tomato revealed that succinic acid exhibited the most significant inhibitory effect against bacterial wilt. Further mechanistic studies demonstrated that its disease resistance function is primarily achieved by recruiting the beneficial bacterium Sphingomonas sp. WX113, and the disease control efficacy of their combined application was significantly superior to that of using either alone (Wang et al., 2025). Research has shown that when salicylaldehyde is used as a fumigant at concentrations ≥0.0001 mol/L, it can effectively inhibit the growth of *Aspergillus flavus* and *Aspergillus parasiticus*. Further evaluation of the effects of 2-hydroxybenzaldehyde on maize growth indicated that, within the concentration range of 0.00005-0.0002 mol/L, this compound had no significant impact on the germination rate, root length, or seedling height of maize seeds, demonstrating that maize exhibits good tolerance to it (Kim et al., 2024). At a concentration of 0.63 mg/mL, boswellic acid not only effectively inhibits the mycelial growth of *Fusarium subglutinans* and *Fusarium semitectum*, but also exhibits no adverse effects on the germination rate and root length of maize seedlings, thus demonstrating favorable crop safety (Seepe et al., 2022). In this study, significant enrichment of succinic acid, 2-hydroxybenzaldehyde, and boswellic acid was observed in the rhizosphere soil of *P. suffruticosa* ‘Luoyang Hong’ after 12 years of continuous cropping (**Figures 2b, c**). We speculate that these compounds may play a role in maintaining the health of the rhizosphere microecological system by inhibiting pathogenic fungi.

On the other hand, typical allelochemicals, such as trans-ferulic acid, and caffeic acid, showed significant accumulation in the roots (Chen et al., 2022; Khattab et al., 2023). Elevated levels of ferulic acid were associated with oxidative stress, cellular dysfunction, and cell death, which inhibit seedling growth (Pergo and Ishii-Iwamoto, 2011). In soybean, treatment with 0.0001 mol/L ferulic acid resulted in increased lignin accumulation in the roots. The excessive cell wall rigidity resulting from lignin deposition restricted cell elongation and ultimately inhibited root growth (Dos Santos et al., 2004). Previous studies have demonstrated that root growth is significantly inhibited when caffeic acid concentrations reach or exceed 0.5 mmol/L. Following uptake, exogenous caffeic acid enters the downstream phenylpropanoid pathway and is catalyzed by 4CL to form caffeoyl-CoA, which subsequently directed into the lignin-specific biosynthetic pathway. This process promotes the polymerization and deposition of lignin monomers, leading to excessive cell wall rigidification that physically restricts cell elongation and root growth (Bubna et al., 2011). Therefore, the accumulation of ferulic acid and caffeic acid in the roots of *P. suffruticosa* ‘Luoyang Hong’ after 42 years of continuous cropping may indicate that root growth was inhibited (**Figures 2e, f**). This phenomenon suggests that reduced microbial degradation, increased environmental stress, or the emergence of autotoxicity (Wacker et al., 1990) at this stage may collectively impair root growth and nutrient uptake.KEGG pathway analysis revealed that the 34 differential metabolites were primarily enriched in pathways such as glycerophospholipid metabolism, starch and sucrose metabolism, and ABC transporters (**Figure 3d**), which aligns with findings from studies on lily’s response to autotoxicity stress (Cheng et al., 2024). The enrichment of these pathways suggests that plants may communicate with rhizosphere microorganisms through mechanisms such as secreting signaling molecules and regulating energy allocation (Phour et al., 2020; Guo et al., 2025).

Furthermore, given the stress of continuous cropping, in cucumber, vanillic acid can reshape the rhizosphere microbial community structure by significantly increasing the abundance of the pathogenic genus *Fusarium* while suppressing the growth of the antagonistic genus *Trichoderma*. (Chen et al., 2018). In peanut, when the concentration of vanillic acid reaches or exceeds 0.5 μmol/g, it directly and significantly inhibits growth indicators such as taproot length, plant height, branch number, and yield, with the inhibitory effect intensifying as the concentration increases. Additionally, vanillic acid indirectly affects nutrient uptake in peanuts by altering the microecological environment, including changes in rhizosphere soil enzyme activities (Tian et al., 2023). Small-molecule peptides also serve as key mediators in plant-microbe interactions and can promote nutrient absorption, such as small peptides produced by plants (SPPs) and the synthetic peptide C-terminally Encoded Peptide 1 (CEP1) (Roy et al., 2022; Tan et al., 2024). This study presents, for the first time, a combined analysis of root metabolites and rhizosphere soil metabolites, revealing that vanillic acid, LysoPC (20:3 (5Z,8Z,11Z)/0:0), and the oligopeptide Leu-Val-Arg-Lys exhibited significant positive correlations in their abundance variations between roots and rhizosphere soil. These results indicate that these metabolites may act as key substances mediating root-soil interactions.

### 4.2 Continuous cropping changed the structure of the soil microbial community

Soil microbial community dysbiosis and functional deterioration are key factors triggering continuous cropping obstacles (Haq et al., 2025; Zhang et al., 2024). The reduction of beneficial bacteria and the impairment of carbon cycling functions within the microbial community following three years of continuous cropping of Codonopsis tangshen have driven the occurrence of continuous cropping obstacles (Xu et al., 2025). During short-term continuous cropping of soybean (7–13 years), beneficial bacterial genera (e.g., *Enterobacter*, *Pseudomonas*, and *Burkholderia*) were depleted, allelopathic autotoxins accumulated, and the microbial interaction network became simplified and fragile, leading to a peak in continuous cropping obstacles at 13 years. However, after long-term continuous cropping (19–25 years), beneficial bacteria including *Bradyrhizobium*, *Pseudomonas*, and *Burkholderia* were significantly enriched, while the relative abundance of pathogenic genera decreased. Concurrently, the microbial interaction network became more complex, and the structure and function of the microbial community approached those of a rotation system, indicating that long-term continuous cropping facilitates the formation of disease-suppressive soil (Liu et al., 2020). *Sphingomonas* enhances stress resistance and promotes growth in plants (Wang et al., 2020), playing a crucial role in reshaping healthy microbial communities (Wang et al., 2024a). *Lysobacter* can reduce crop diseases caused by pathogens (Puopolo et al., 2018; Ji et al., 2008), a finding that has been confirmed in crops such as soybean (Yu et al., 2020), rice (Yu et al., 2020), and grape (Puopolo et al., 2014). *Nitrospira* is a key driver of the nitrogen cycle, playing an essential role in nitrification by oxidizing nitrite to nitrate (Zhao et al., 2025b). After 12 years of continuous cropping, the genera *Sphingomonas*, *Lysobacter*, and *Nitrospira* were found to be enriched (**Figure 5a**). This enrichment indicates that the microbial community maintained a sound ecological function in the early stages of continuous cropping. However, after 22 years of continuous cropping, there was a significantly decrease in the abundance of beneficial bacterial genera, including *Sphingomonas*, *Novosphingobium*, and *Solirubrobacter* (**Figure 5b**). *Novosphingobium*contributes to soil nutrient cycling by degrading a wide range of organic pollutants and complex carbon compounds (Segura et al., 2021). Similarily, *Solirubrobacter* plays a key role in maintaining soil health and promoting nutrient cycling (Qiao et al., 2024; Jara-Servin et al., 2024). This decline suggests that the corresponding soil ecological functions became weakened or disordered after 22 years of continuous cropping of *P. suffruticosa* ‘Luoyang Hong’, which aligns with the findings of Wang et al. (2012). Notably, after 34 years of continuous cropping, microbial genera associated with the degradation of complex organic matter, such as *Steroidobacter* and *Gaiella*, were significantly enriched (Fahrbach et al., 2008; Liu, L. et al., 2023). This finding implies that the soil microbial community of *P. suffruticosa* ‘Luoyang Hong’ may have evolved a self-repair strategy after 34 years of continuous cropping, (**Figure 5c**). Subsequently, after 42 years of continuous cropping, a recurrence of beneficial genera, including *Sphingomonas*, *Solirubrobacter*, *Nocardioides*, and *Blastococcus* was observed (**Figure 5d**). *Nocardioides* is a group of microorganisms (Castro et al., 2018) with significant application value in environmental remediation, as they are capable of efficiently degrading a variety of recalcitrant organic pollutants (Ma et al., 2023). This suggests a potential partial recovery of rhizosphere microecological function. The phenomenon of gradual recovery of the soil microbial community after long-term continuous cropping has been documented in soybean and tobacco (Liu et al., 2020; Li et al., 2024b; Xuan et al., 2024). However, compared with the SY12 group, the abundances of Lysobacter, Sphingomonas, and Nitrososphaera were decreased in the SY42 group (**Figure 5d**). This observation may indicate that although the soil exhibits a certain degree of self-recovery, its overall ecological function still demonstrates a trend of degradation (Ma L. et al., 2024). In the absence of appropriate remedial measures, the soil community may face further degradation. This understanding has significant implications for the development of control strategies aimed at addressing the challenges associated with continuous cropping of tree peony.

### 4.3 Changes in soil microbial function and soil nutrients

Based on the analysis of the relative abundance of KEGG Level 2 pathways the shifts in the potential functional profiles of the soil microbial community and its community structure under long-term continuous cropping (0–42 years) of *Paeonia suffruticosa* ‘Luoyang Hong’ exhibited similar trends (**Figure 5**, **6**). This further supports the observation that the 22nd year of continuous cropping represents a stage of soil microbial community imbalance, with evidence of partial potential functional reconstruction detected in the 42nd year of continuous cropping.

From the perspective of soil nutrients, during the long-term cultivation of *P. suffruticosa* ‘Luoyang Hong’ (Tab 1), the contents of soil total potassium (TK) and available potassium (AK) did not show deficiency characteristics, indicating that the potassium supply in this cultivation system was sufficient. It is noteworthy that the soil organic matter (SOM) content in all continuous cropping groups was significantly lower than in the control group, but it showed a slow upward trend during the 12- to 42-year of continuous cropping, which may be related to the continuous input and gradual accumulation of plant root exudates and litter over many years of cultivation (Song et al., 2025).

Long-term cultivation of *P. suffruticosa* ‘Luoyang Hong’ tree peony also had a significant impact on soil nitrogen and phosphorus nutrients and their related microbial functional genes (**Figure 7a**). With increasing planting years, soil total nitrogen (TN) and ammonium nitrogen (NH_4_^+^-N) significantly decreased in the early stages (12-, 22-year) (Mu et al., 2024), while nitrate nitrogen (NO_3_^--^N) peaked at 34 years. Correspondingly, among the nitrogen cycle-related functional genes, the organic nitrogen mineralization gene (*ureC*) was significantly higher than the control in all 12–42 years plantings; the assimilatory nitrate reductase gene (*nasA*) continuously increased with the extension of planting years; and the nitrogen fixation genes (*nifK, nifH*) peaked in abundance at 34 years, indicating that microbial-mediated nitrogen transformation functions produced a positive adaptive response to the stress of continuous cropping.

In terms of phosphorus, TP significantly decreased in the early stages of continuous cropping (12-, 22-year), indicating phosphorus depletion due to plant uptake and soil fixation (**Figure 7b**). At this time, rhizosphere microorganisms promoted the conversion of organic phosphorus to available phosphorus by up-regulating the organic phosphorus mineralization gene *appA*; by the middle and late stages (22-, 34-, 42-year), the abundance of the inorganic phosphorus solubilizing gene (*gcd*) also increased, alleviating phosphorus stress through a dual compensation mechanism of organic phosphorus mineralization and inorganic phosphorus solubilization. The peak in available phosphorus (AP) at 34 years triggered a significant down-regulation of the phosphorus starvation response regulatory genes (*phoB*, *phoR*, *spoT*), forming a negative feedback loop characterized by increased phosphorus availability, a weakened cellular starvation signal, and consequently, a reduction in regulatory gene abundance.

The above results indicate that under long-term continuous cropping conditions, rhizosphere microorganisms can actively respond to changes in soil nitrogen and phosphorus nutrient status by adjusting community structure and functional gene expression, thereby maintaining and restoring soil nutrient cycling functions to a certain extent, reflecting the ecological adaptation mechanism of microorganisms to continuous cropping stress (Lang et al., 2025).

### 4.4 Interaction between metabolites and the soil microbial community

Root exudates serve as substrates or signaling molecules for microorganisms, thereby altering and shaping the composition of the rhizosphere microbial community (Shafi and Shahid, 2025). Flavonoids attract Lysobacter soli by stimulating its twitching motility and spermidine biosynthesis, which in turn enhances vitamin accumulation in fruits across various tomato cultivars and soil types (Shafi and Shahid, 2025). Organic acids present in banana root exudates actively recruit *Bacillus amyloliquefaciens* strain NJN-6 to colonize its roots thereby imoroving the plant’s disease resistance (Yuan et al., 2015). Root exudates not only provide ecological niches for beneficial microorganisms but may also enrich or chemoattract pathogens, potentially inducing soil-borne diseases and exacerbating the challenges associated with continuous cropping. For instance, allelochemicals such as catalpol and acteoside secreted by Rehmannia glutinosa under continuous cropping promote the colonization of pathogens in the rhizosphere, directly contributing to the outbreak of root rot (Yuan et al., 2024). Fumaric acid secreted by tobacco roots can induce *Ralstonia solanacearum* to colonize and infect the roots, a key factor in the outbreak of tobacco bacterial wilt (Li et al., 2017). Cinnamic acid, an autotoxic allelochemical found in cucumber roots, stimulates the colonization and pathogenic ability of *Fusarium oxysporum* within the root system, significantly increasing the incidence of *Fusarium* wilt in cucumber (Ye et al., 2004). N,N-Dimethyldodecylamine N-oxide is an amphoteric surfactant, structurally similar to N-Dodecyl-N,N-bis(3-(aldonamido)-propyl)amine-N-oxides, and such substances usually have antibacterial and emulsifying properties (Piasecki et al., 2001). The results of this study revealed that this substance exhibited significant positive correlations with several beneficial microbial genera, including *Solirubrobacter*, *Sphingosinicella*, and *Blastococcus* (**Figure 8c**), suggesting its potential role as a key signaling molecule employed by plant roots to recruit beneficial microorganisms and maintain rhizosphere microecological health.

A significant positive correlation was also found linking *Phenylobacterium* and oleanolic acid (**Figure 8c**), a strictly aerobic, Gram-negative bacterium capable of degrading benzene rings and a pentacyclic triterpenoid compound, respectively. Oleanolic acid may act as an allelochemical that provides a specific carbon source or growth signal for this bacterium, promoting its colonization and proliferation in the rhizosphere (Wang et al., 2014).

In addition, the short peptide Leu-Val-Arg-Lys and the phospholipid PG (22:6/0:0) also showed a significant positive correlation with *Phenylobacterium* (**Figure 8c**). These metabolites may be involved in the metabolic processes or cell membrane composition of this bacterium, closely related to its growth and metabolic activities (Tan et al., 2024; Read et al., 2003).

By uncovering how the *P. suffruticosa* ‘Luoyang Hong’ actively manipulates its rhizosphere microbiome through root-derived metabolites, this study provides molecular-level insights into the regulatory mechanisms of this micro-ecosystem under continuous cropping conditions (Deng et al., 2023). Overall, the research findings on the relationship between metabolites and microbial communities provide valuable guidance for regulating the rhizosphere microbial community through biotechnological approaches (e.g., microbial fertilizer), thus offering a theoretical basis for overcoming the continuous cropping obstacles of *P. suffruticosa* ‘Luoyang Hong’ (Zhao et al., 2025a).

## 5 Conclusion

This study systematically characterized the compositional and abundance features of root metabolites in *P. suffruticosa* ‘Luoyang Hong’ under long-term continuous cropping conditions, clarifying the dynamic succession patterns of its rhizosphere microecology. Metabolites including Leu-Val-Arg-Lys, 3-hydroxybenzoic acid and oleanolic acid may play a pivotal role in the interaction between the roots of *P. suffruticosa* ‘Luoyang Hong’ and its rhizosphere soil. With the extension of continuous cropping years, the rhizosphere microbial community of *P. suffruticosa* ‘Luoyang Hong’ exhibited a dynamic succession characteristic of spiral decline: an initial enrichment of beneficial microbial communities, followed by dysbiosis of the microbial community structure at the middle stage, and a partial recovery of community functions at the late stage, all within an overall degenerative trend. Through integrated metagenomic and metabolomic analyses, this study elucidates the effects of continuous cropping on the ecological functions of the rhizosphere microbial community in *P. suffruticosa* ‘Luoyang Hong’. It provides an important theoretical basis for subsequent studies on the fine-scale succession of microbial communities and the verification of the ecological functions of key metabolites.

## Data Availability

The original contributions presented in the study are included in the article/**Supplementary Material**. Further inquiries can be directed to the corresponding author.
