# Supplementary material for: Metagenomic and metabolomic insights into the rhizosphere of Paeonia suffruticosa ‘Luoyang Hong’ across a continuous cropping chronosequence
Source: Front Plant Sci. 2026 May 12;17:1754999. doi: 10.3389/fpls.2026.1754999 (PMC13201231; doi:10.3389/fpls.2026.1754999)
Supplement: Supplementary file 1 [file Table1.docx]

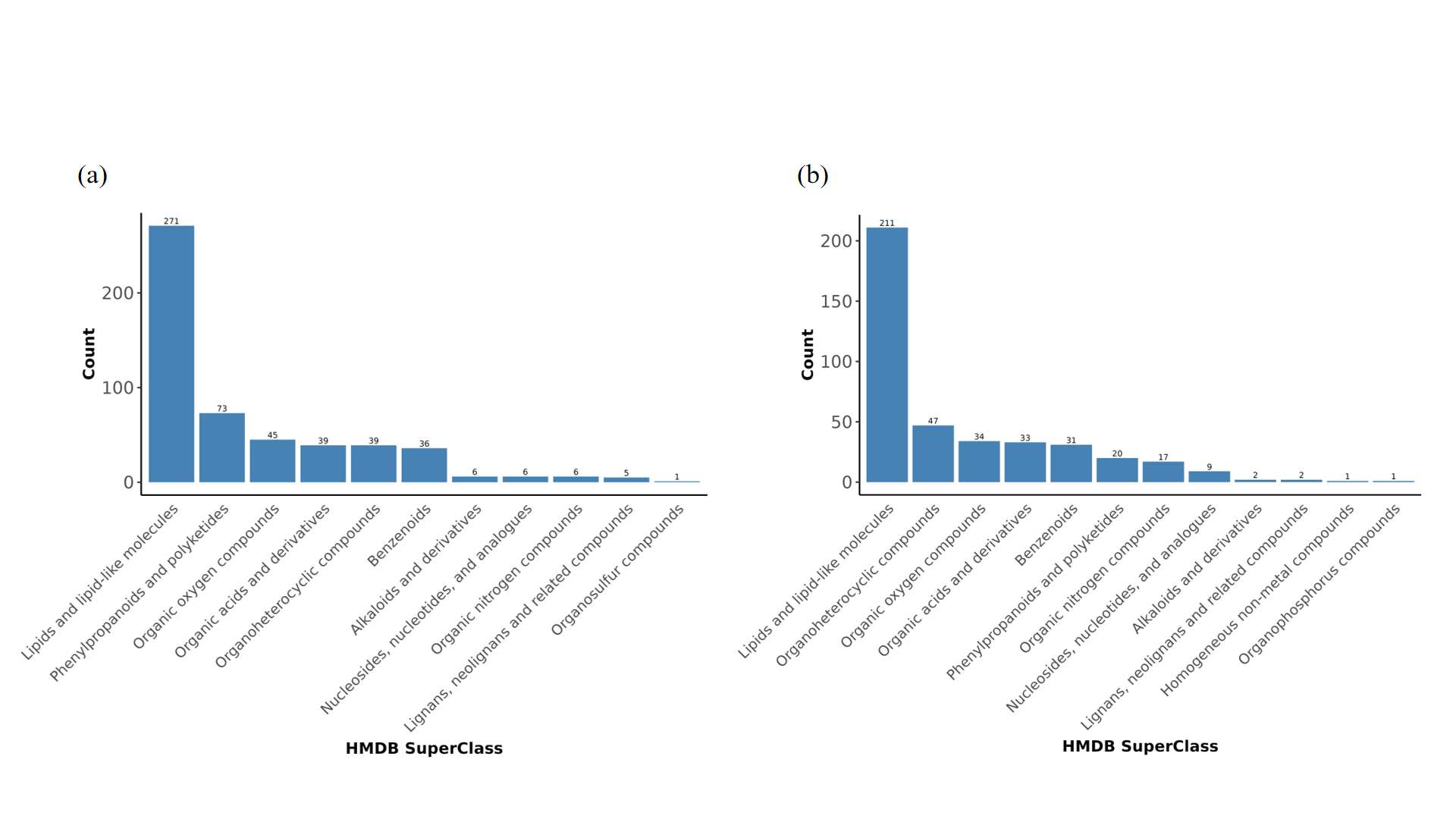
**Supplementary Figure_1.** Superclass of Metabolites. **(a)** Metabolic superclasses identified in the root system of *Paeonia suffruticosa* ‘Luoyang Hong’; **(b)** Metabolic superclasses identified in the rhizosphere soil of *Paeonia suffruticosa* ‘Luoyang Hong’.


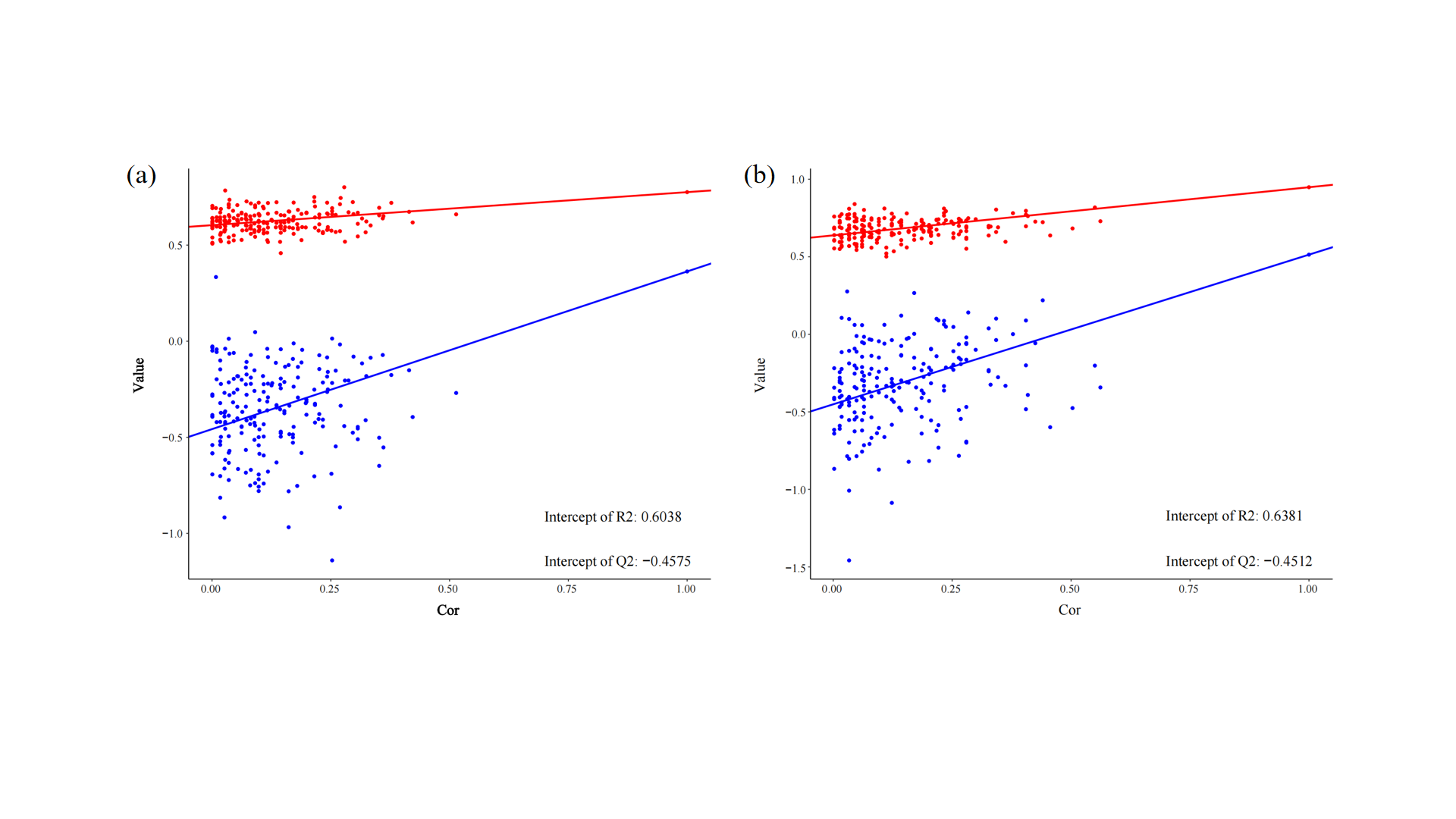
**Supplementary Figure_2.** **(a)** Permutation test plot of rhizosphere soil metabolites; **(b)** Permutation test plot of root metabolites.

Note: The R^2^ regression line is in red, and the Q^2^ regression line is in blue.


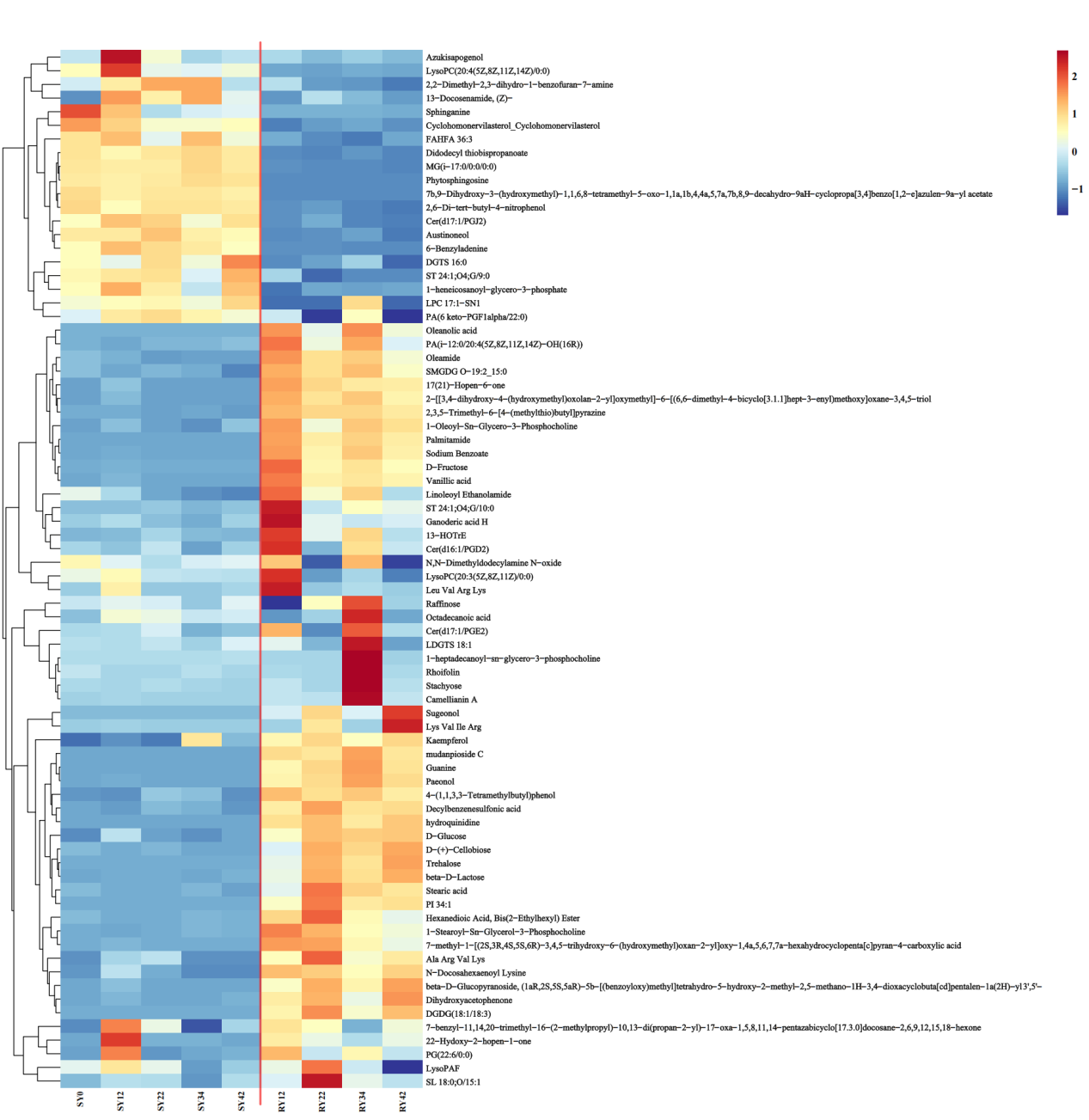


**Supplementary Figure_3.** Abundance changes of the 76 shared metabolites in soil vs. root.


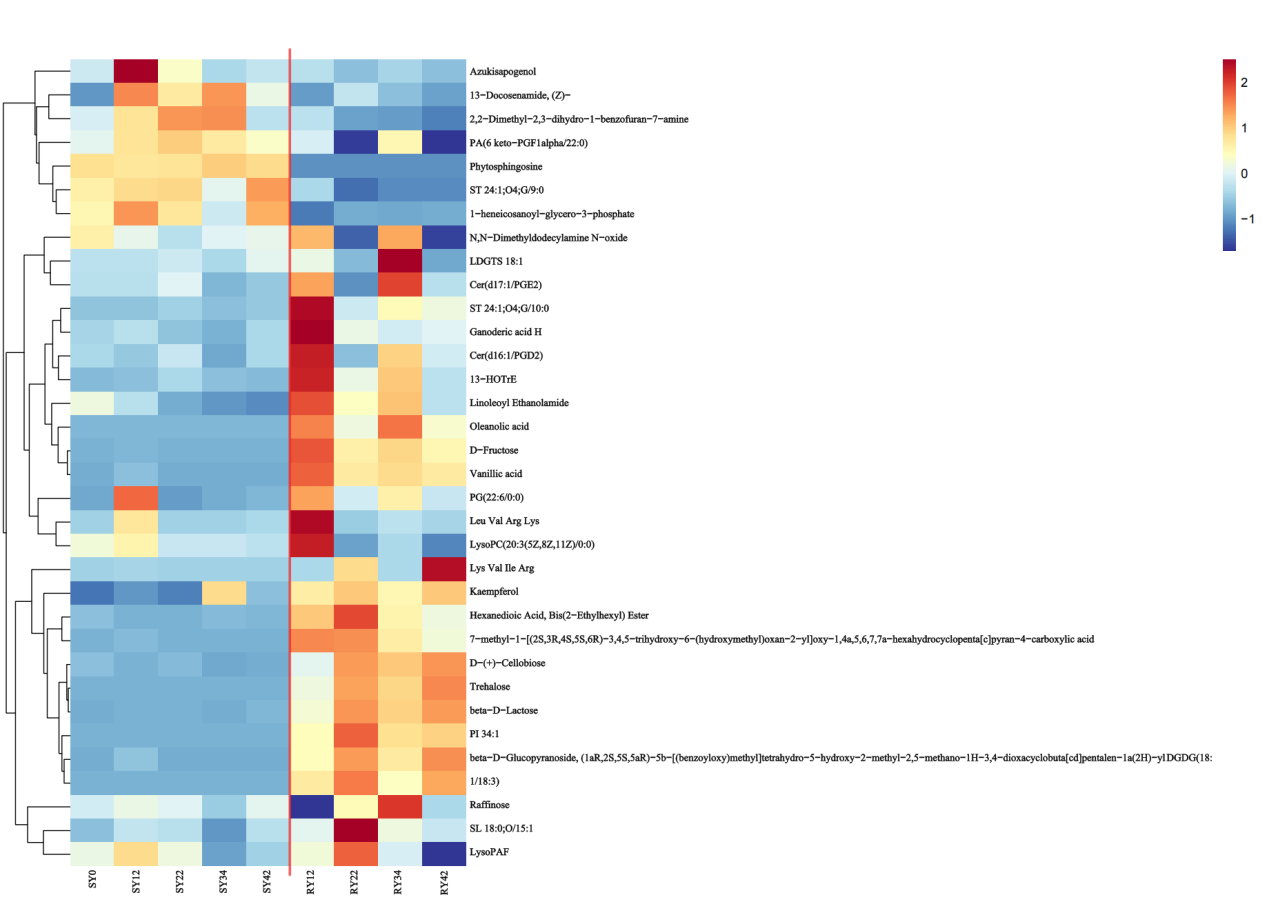


**Supplementary Figure_4.** Abundance changes of the 34 differentially abundant shared metabolites in soil vs. root.


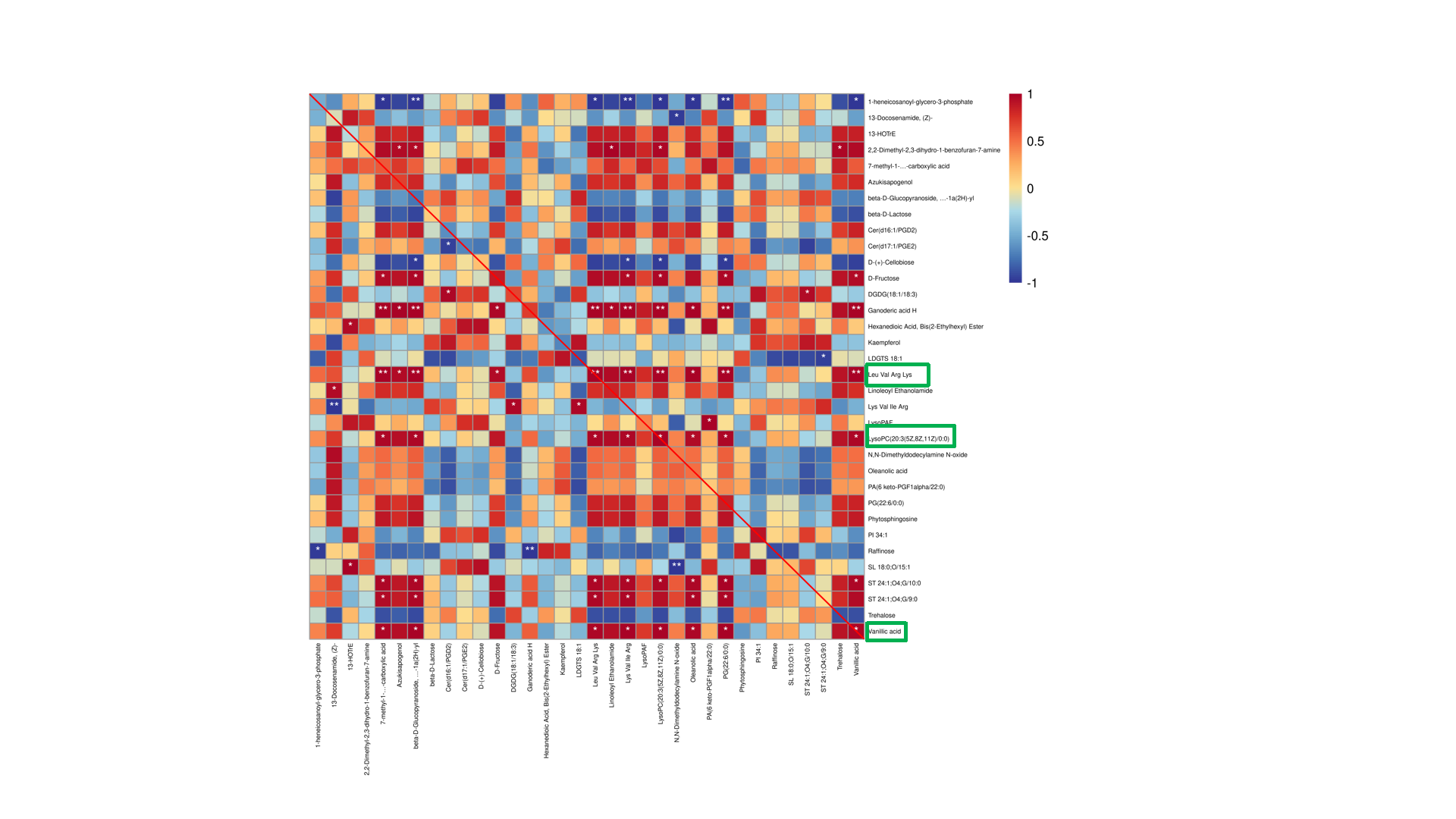


**Supplementary Figure_5.** Correlation analysis for abundance changes of the 34 differential shared metabolites in soil vs. root.


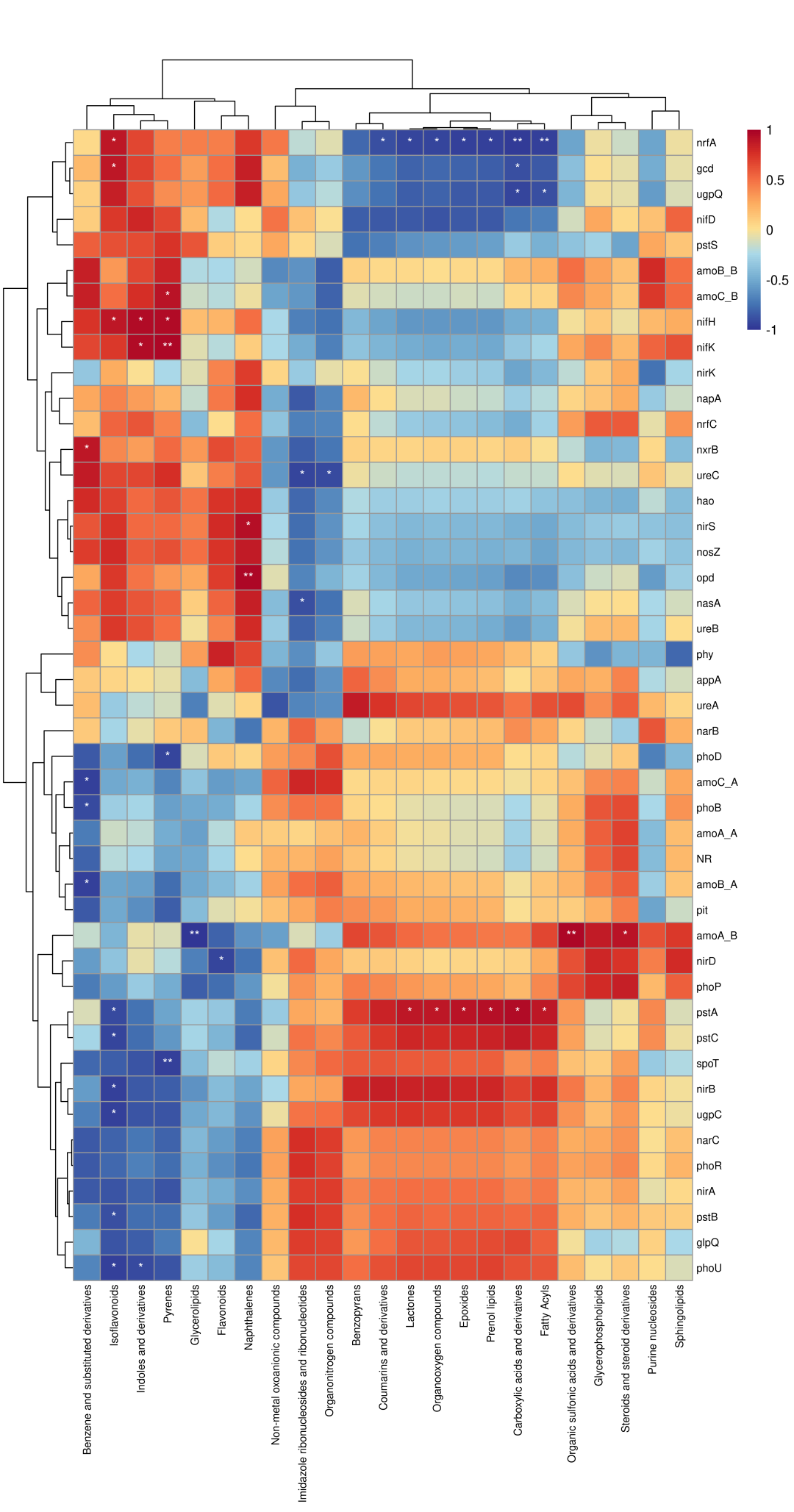


**Supplementary Figure_6.** Correlation analysis between nitrogen and phosphorus functional genes and classes of differential metabolites in the rhizosphere soil.


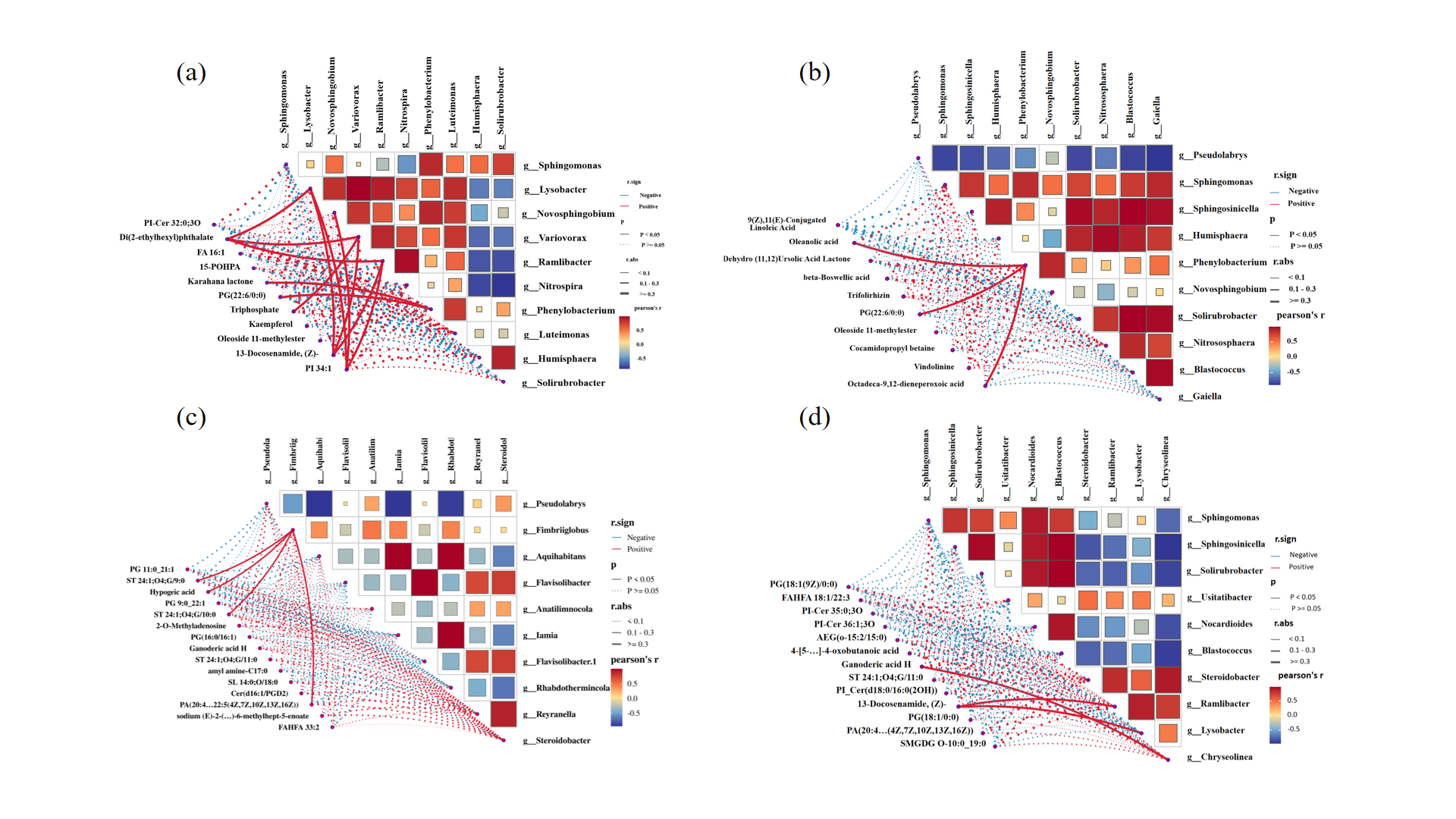


**Figure_7.** Correlation analysis. **(a)**Correlation between Differential Metabolites and Differential Microbial Genera in SY12 vs SY0; **(b)** Correlation between Differential Metabolites and Differential Microbial Genera in SY22 vs SY12; **(c)** Correlation between Differential Metabolites and Differential Microbial Genera in SY34 vs SY22; **(d)** Correlation between Differential Metabolites and Differential Microbial Genera in SY42 vs SY34.

Note: Pearson correlation test was used (*P*≤0.05). In the heatmap on the right, the colors of the squares represent the strength of correlation between metabolite groups: the redder the color, the stronger the positive correlation; the bluer the color, the stronger the negative correlation. The network diagram in the bottom left corner shows the correlation analysis results between differential species and differential metabolites, where the thickness of the lines represents the r-value (the thicker the line, the stronger the correlation), red lines indicate positive correlation, blue lines indicate negative correlation, solid lines indicate *P*≤0.05, and dashed lines indicate *P*>0.05. *indicates*P*≤0.05, and **indicates *P*≤0.01
